# Gut microbiome interacts with pregnancy hormone metabolites in gestational diabetes mellitus

**DOI:** 10.3389/fmicb.2023.1175065

**Published:** 2023-07-10

**Authors:** Xuejing Lyu, Shaona Wang, Jiaxin Zhong, Lingzhu Cai, Yanhui Zheng, Ying Zhou, Ying Zhou, Qionghua Chen, Qiyuan Li

**Affiliations:** ^1^Clinical Medical Research Center for Obstetrics and Gynecology Diseases of Fujian Province, Laboratory of Research and Diagnosis of Gynecological Diseases of Xiamen City, Department of Obstetrics and Gynecology, The First Affiliated Hospital of Xiamen University, School of Medicine, Xiamen University, Xiamen, China; ^2^School of Medicine, National Institute of Data Science in Health and Medicine, Xiamen University, Xiamen, China; ^3^The Third Clinical Medical College, Fujian Medical University, Fuzhou, China; ^4^Department of Women’s Health, Xiamen Haicang District Maternity and Child Health Care Hospital, Xiamen, China; ^5^Department of Pediatrics, The First Affiliated Hospital of Xiamen University, Xiamen, China

**Keywords:** gestational diabetes mellitus, gut microbiota, hormone metabolism, time series, metabolic biomarker

## Abstract

**Introduction:**

Change in the composition of intestinal microbiota is associated with metabolic disorders such as gestational diabetes mellitus (GDM).

**Methods:**

To understand how the microbiota impacts the development of gestational diabetes mellitus, we profiled the intestinal microbiome of 54 pregnant women, including 27 GDM subjects, by employing 16S rRNA gene sequencing. Additionally, we conducted targeted metabolomics assays to validate the identified pathways with overrepresented metabolites.

**Results:**

We evaluated the patterns of changing abundances of operational taxonomic units (OTU) between GDM and the healthy counterparts over three timepoints. Based on the significant OTUs, we inferred 132 significantly altered metabolic pathways in GDM. And identified two overrepresented metabolites of pregnancy hormone, butyrate and mevalonate, as potential intermediary metabolites of intestinal microbiota in GDM. Finally, we validated the impacts of the intestinal microbiota on GDM by demonstrating consistent changes of the serum levels of progesterone, estradiol, butyrate, and mevalonate in an independent cohort.

**Discussion:**

Our findings confirm that alterations in the microbiota play a role in the development of GDM by impacting the metabolism of pregnancy hormones. This provides a novel perspective on the pathogenesis of GDM and introduces potential biomarkers that can be used for early diagnosis and prevention of the disease.

## 1. Introduction

Gestational diabetes mellitus (GDM) is a common metabolic disorder responsible for numerous adverse maternal and neonatal outcomes(Beucher et al., 2010; Ismail et al., 2011; Catalano et al., 2012). Primigravidae diagnosed with GDM are at a greater risk of GDM (38.19%) in the second pregnancy than their healthy counterparts (3.52%) (Gomez-Arango et al., 2016). The incidence rate of GDM worldwide is 17.8% (with a range of 9.3–25.5%) according to Sacks et al. (2012). However, controlling GDM is challenging due to lack of screening, difficulty in gestational weight management, and safety concerns over the use of long-term medications (Matt, 2020).

Abnormalities in the reprogramming of metabolic processes cause GDM. The latter is characterized by insulin resistance displayed in the second or third trimester, attributed to increased levels of certain pregnancy and placental hormones and pro-inflammatory factors (Mor and Cardenas, 2010; Kampmann et al., 2019). While most of the GDM subjects’ blood glucose normalizes right after birth, about 20–50% develop type II diabetes during the 5 years postpartum (Kim et al., 2002).

Pregnancy hormones block insulin sensitivity hence partially causing GDM (Root-Bernstein et al., 2014). Notably, during pregnancy, hormones’ levels vary substantially among individuals (Kumar and Magon, 2012). Such variations are associated with decreased insulin sensitivity (Lain and Catalano, 2007). Interestingly, hormone levels in pregnancy can be regulated by microbiota and profoundly impact the endocrine system resulting in metabolic disorders (Koren et al., 2012; Neuman et al., 2015; Liu et al., 2019). Furthermore, it has been observed that altered gut microbiota constructions, the host’s hormone levels, and physiological changes during pregnancy are strongly correlated (Neuman et al., 2015).

The hormonal changes during pregnancy are known to interact with the gut microbiota (Neuman et al., 2015; Qi et al., 2021). Recent studies have shown that these interactions resulting in altered hormone metabolism, which directly links to the development of GDM. Specifically, metabolites such as short-chain fatty acids (SCFAs) and lipopolysaccharides (LPS), are produced by gut bacteria during the fermentation of dietary fibers (Gomez-Arango et al., 2016). Increased levels of SCFAs have been shown to reduce insulin resistance and improve glucose metabolism in pregnancy (Wong et al., 2006; Yang et al., 2015). However, high level of SCFAs is also controversially reported for association with increased risk of GDM (Hasain et al., 2020). On the other hand, elevated levels of bacterial LPS have been associated with inflammation and insulin resistance, while butyrate has been shown to have an inhibitory effect on LPS production (Roy et al., 2020).

However, the intermediary metabolites mediating microbiota’s impact on pregnancy hormones and GDM remain unknown. Moreover, most prior studies on GDM’s microbiota are based on cross-sectional data with a case–control setting. Notably, changes in the gut microbiota of GDM subjects are often confounded with individual variation due to limited sample size.

In this study, we hypothesize a metabolic mediation between gut microbiota and pregnancy hormones’ activity, affecting insulin and glucose metabolism during pregnancy. We profiled the gut microbiome at three different time points during pregnancy in both GDM subjects and healthy pregnant women. Our data suggest that gut microbiota is involved in GDM’s pathogenesis by influencing the biogenesis of steroid hormones through butanoate and mevalonate’s metabolic pathways.

## 2. Materials and methods

### 2.1. Subject recruitment and inclusion criteria

**Figure 1:**
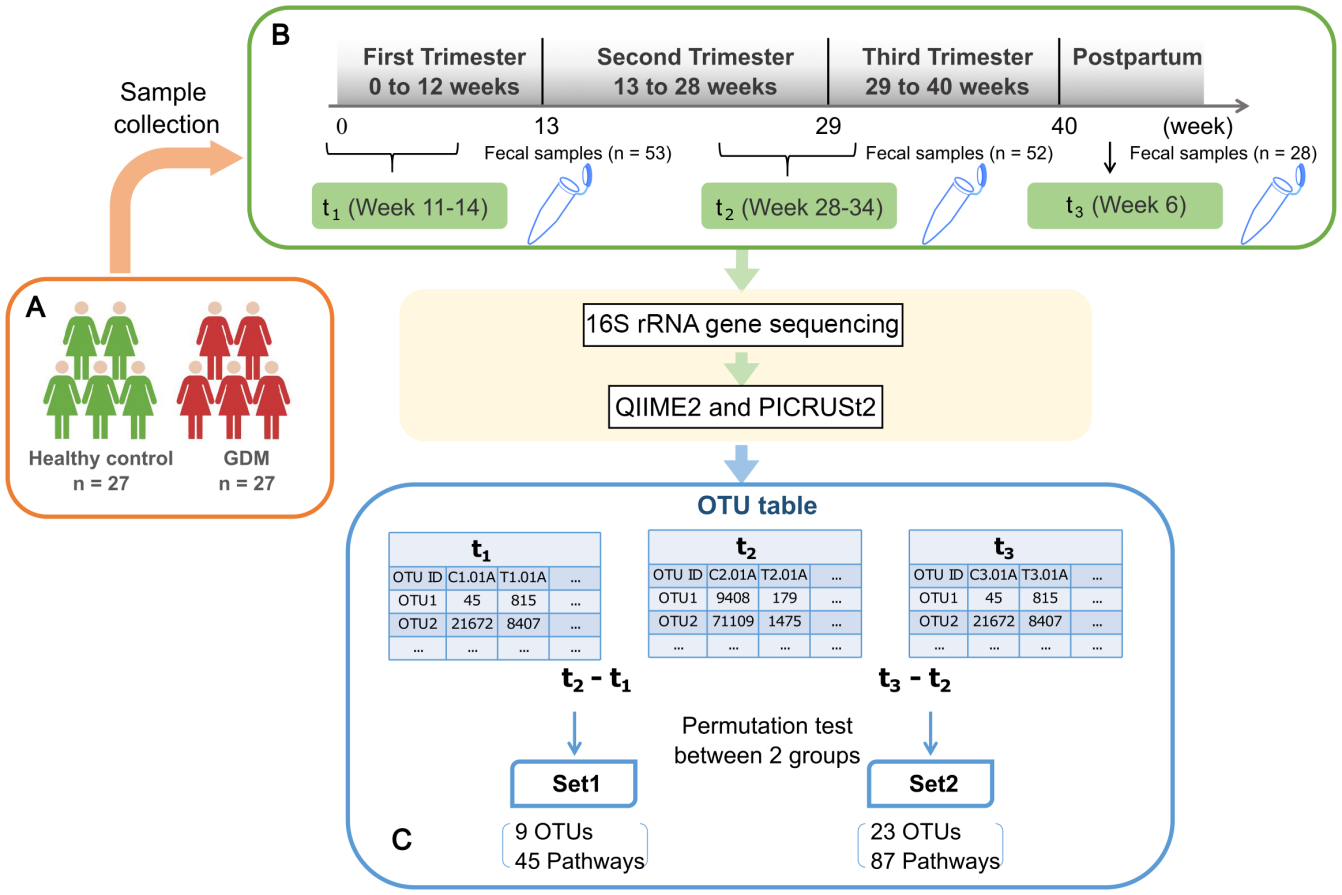
Schematic view of the study design. **(A)** 27 healthy pregnant women and 27 GDM subjects are enrolled in the study. Subject enrolment criteria were as follows: natural conception and singleton pregnancy; age below forty years; BMI pre-pregnancy between 17–28 (kg/m2); and gestational age below 14 weeks. Exclusion criteria were diabetes pre-pregnancy, history of GDM, polycystic ovary syndrome, hypertension, depression, pregnancy-related diseases, infectious diseases, blood system diseases, and immune system diseases. Additionally, included subjects had no recent serious intestinal diseases, antibiotics, hormones, or probiotic treatment within one month prior to fecal sampling. **(B)** Fecal samples were collected from enrolled subjects at three time points as shown in the green rectangles, including early (t_1_) and late (t_2_) stage of pregnancy, and postpartum(t_3_). After 16 s rRNA gene sequencing, the abundances of operational taxonomic units (OTU) are inferred using QIIME2. **(C)** The differences of microbial profiles between two time points for each subject were calculated by log-ratios of t_2_ vs. t_1_ and t_3_ vs. t_2_, respectively. The results were tested based on 1,000-fold permutation to obtain significant OTUs, namely Set1-OTUs, Set2-OTUs. Then we inferred the activities of metabolic pathways and performed permutation test in the same way to obtain Set1-pathways and Set2-pathways.

We selected 54 pregnant women admitted to the obstetrics department of the First Affiliated Hospital of Xiamen University between March 2018 and March 2019, who underwent regular prenatal check-ups, and gave birth in the hospital (Figure 1A). The clinical research protocols were approved by the Ethics Committee of the First Affiliated Hospital of Xiamen University (reference number KY2022-033). All volunteers signed an informed consent according to protocol. Enrollment and exclusion criteria are summarized in Figure 1 caption.

All enrolled subjects received an oral glucose tolerance test (OGTT) at 24–28 weeks. After 3 days of a regular diet with at least 150 g daily carbohydrate intake, the subjects underwent 8-h fasting and were then examined. A standard 2-h OGTT was conducted and gestational diabetes mellitus (GDM) diagnosis was performed based on the International Association of Diabetes and Pregnancy Study Groups’ diagnostic criteria. Diagnostic cut-off values of fasting, 1-h, and 2-h plasma glucose levels were 5.1, 10.0, and 8.5 mmol/L. Women whose blood glucose values met or exceeded the above criteria at either time received a GDM diagnosis (GDM subjects). Age and body mass index (BMI)-matching pregnant women who tested negative were considered controls.

For further validation using a targeted metabolomics assay, peripheral blood samples were collected from an independent cohort of 28 GDM subjects and controls (Supplementary Table S1).

### 2.2. Microbiome sample collection

Nurses sampled stool specimens at three time-points during pregnancy: t_1_ (first trimester, 11.85 ± 1.05 weeks), t_2_ (third trimester, 30.50 ± 2.59 weeks), and t_3_ (6–8 weeks postpartum, Figure 1B; Table 1). To ensure the sampling accurately reflected the human intestinal flora, we injected 5 mL of saline into the anus and repeated suction. We then extracted the fecal suspension from the flushed rectum. The suspension was then injected into 1.5 mL Eppendorf (EP) tubes. We collected 2–5 specimens per subject/time point, stored them on ice and quickly transferred them to −80°C. Dry ice transportation was used to reduce oxidation risk.

**Table 1:** The clinical characteristics of enrolled subjects, early pregnant test, and plasma measurements of the study cohort.

		GDM (*n* = 27)	Control (*n* = 27)	*p* value	FDR
Sample information	Age (year)	30.96 ± 3.79	29.80 ± 4.10	0.2945	0.5890
	Pre-BMI (kg/m^2^)	20.67 ± 1.91	20.49 ± 2.01	0.7577	0.9300
	Total weight gain (kg)	11.05 ± 2.97	13.44 ± 3.88	0.0151	0.1208
Early preganacy test	FGB (mmol/L)	4.84 ± 0.42	4.89 ± 0.33	0.598	0.9300
(t_1_)	HB (g/L)	123.00 ± 9.64	129.11 ± 10.05	0.028	0.1493
	ALT (U/L)	19.12 ± 14.65	17.38 ± 5.49	0.2528	0.5778
	AST (U/L)	16.78 ± 3.84	18.96 ± 12.15	0.7547	0.9300
	Urea (mmol/L)	3.18 ± 0.68	3.16 ± 0.78	0.93	0.9300
	Creatine (μmol/L)	49.54 ± 10.74	50.26 ± 9.95	0.7997	0.9300
Plasma measurements	HbA1c (%)	5.41 ± 0.43	5.01 ± 0.23	0.0012	0.0192
(t_2_)	P (ng/mL)	35.99 ± 10.31	42.53 ± 13.31	0.0583	0.2332
	TC (mmol/L)	5.87 ± 1.03	5.98 ± 1.51	0.8289	0.9300
	TG (mmol/L)	2.73 ± 0.95	2.35 ± 0.76	0.2302	0.5778
	HDL-C (mmol/L)	1.77 ± 0.31	1.80 ± 0.53	0.8739	0.9300
	LDL-C (mmol/L)	2.66 ± 0.61	3.09 ± 0.65	0.0864	0.2765
	TSH (mIU/mL)	1.57 ± 0.61	1.81 ± 1.69	0.7135	0.9300

*p* value adjusted by FDR.

For all enrolled subjects, we collected extensive clinical information, documented in Table 1.

### 2.3. Microbiome analysis

#### 2.3.1. 16S rRNA gene sequencing and pre-processing

All specimens were sent to BGI Co. Ltd. for database construction and sequencing. Qualifying genomic DNA samples were selected, the variable region 4 (V4) of the 16S ribosomal RNA (rRNA) gene was used as an amplicon, and sequencing was conducted on the HiSeq platform. Genomic DNA samples of 30 ng were extracted and the corresponding fusion primers were configured for PCR amplification. The amplified products were purified using Agencourt AMPure XP magnetic beads and eluted in Elution Buffer. The resulting library was labeled and prepared for sequencing by using the Agilent 2,100 Bioanalyzer to detect fragment size and concentration. Qualified libraries were sequenced on the HiSeq platform based on the size of the inserted fragments. First, the original sequencing data was filtered for reads that were adaptor-contaminated, those containing N and low-complexity ones. Barcodes were removed before downstream analysis.

#### 2.3.2. 16S rRNA gene sequencing data analysis

Raw sequence data were demultiplexed and quality-controlled using Quantitative Insights into Microbial Ecology (QIIME2, RRID:SCR_021258). QIIME2 plugin DADA2 was used to denoise and produce operational taxonomic units (OTUs) at 100% similarity. We employed the Greengenes database (RRID:SCR_002830) as a reference to annotate OTUs. Taxonomic classification of marker-gene amplicon sequence was done using the sklearn classifier (q2-feature-classifier, https://github.com/qiime2/q2-feature-classifier). Alpha diversity was assessed based on observed species, Shannon index, and Simpson index using the phyloseq package in R-3.6.3. Beta diversity between GDM subjects and controls at different time-points was measured using principal component analysis (PCA) and linear discriminat analysis (LDA). The LDA analysis was done using ‘lda’ function in ‘MASS’ package, and visualized by‘ggord’ function in ‘ggord’ package. We also carried out LEfSe (Linear discriminant analysis Effect Size) analysis on Galaxy web application (Segata et al., 2011) to reveal the differentially abundant OTUs. The data were split by timepoint (t_1_, t_2_, t_3_), grouped by condition (GDM or normal).

#### 2.3.3. Significant OTUs between GDM and healthy controls

We used log-ratio to measure each OTU’s difference of abundance between two adjacent time-points (Figure 1C). Then, each OTU’s significance of the difference between GDM subjects and controls was evaluated by 1,000-round permutation test implemented using the “ez” package in R (R code is available on https://github.com/xmbd/Gestational_diabetes_mellitus).

### 2.4. Metabolic pathway prediction and analysis

Activities of microbial metabolic pathways were predicted using the PICRUSt2 (Phylogenetic Investigation of Communities by Reconstruction of Unobserved States) plugin in QIIME2^1^ based on the OTU table obtained from QIIME2. MetaCyc database (Caspi et al., 2020) was used as a reference for the annotation and identification of related metabolites in these pathways. Significantly altered pathways in GDM were called similarly based on permutation testing. We then performed K-means clustering on Set1- and Set2- pathways based on activity changes between GDM subjects and controls. Subsequently, the outliers were filtered out in Set2 (Principal component 2 < −5 or > 5). For each cluster of pathways, we retrieved all the compounds from the MetaCyc database (Caspi et al., 2020). Then we ranked all the metabolites based on occurrence frequency, maintaining the recurrent ones (*n* > 1). Next, we compared the most recurrent metabolites with those in known hormone-metabolic pathways (Holstein and Hohl, 2004; Heuston et al., 2012; Vital et al., 2014). Finally, we defined each cluster’s representative pathways (Table 2) based on the enrichment of recurrent metabolites (Supplementary Table S4).

**Table 2:** The representative microbial metabolic pathways in each cluster corresponding to the highly recurrent metabolites (butanoate and mevalonate) in Set1- and Set2-pathways.

		PWYID	Cluster	*p* value	Pathway name	Metabolites
**Set1**	Butanoate pathways	P163-PWY	2	0.0012	L-lysine fermentation to acetate and butanoate	acetate, acetoacetyl-CoA, butanoate, acetyl phosphate, butanoyl-CoA, crotonyl-CoA, acetoacetate
		PWY-5676	2	0.0172	acetyl-CoA fermentation to butanoate II	acetate, acetoacetyl-CoA, butanoate, acetyl phosphate, butanoyl-CoA, crotonyl-CoA
		P162-PWY	2	0.0424	L-glutamate degradation V (via hydroxyglutarate)	acetate, acetoacetyl-CoA, butanoate, butanoyl-CoA, crotonyl-CoA
	Mevalonate pathways	NONMEVIPP-PWY	2	0.0335	methylerythritol phosphate pathway I	DMAPP, MEP
		PWY-7560	2	0.0335	methylerythritol phosphate pathway II	DMAPP, MEP
		PWY-5121	2	0.028	superpathway of geranylgeranyl diphosphate biosynthesis II (via MEP)	DMAPP, IPP, MEP, GPP, GGPP
		PWY-7392	2	0.0305	Engineered Pathway: taxadiene biosynthesis (engineered)	DMAPP, MEP, GPP, GGPP
**Set2**	Butanoate pathways	PWY-6590	3	0.0372	superpathway of *Clostridium acetobutylicum* acidogenic fermentation	acetate, acetoacetyl-CoA, butanoate, acetyl phosphate, butanoyl-CoA, crotonyl-CoA
		CENTFERM-PWY	3	0.0373	pyruvate fermentation to butanoate	acetoacetyl-CoA, butanoate, butanoyl-CoA, butanoyl phosphate, crotonyl-CoA
	Mevalonate pathways	PWY-922	3	0.0203	mevalonate pathway I (eukaryotes and bacteria)	acetyl-CoA, DMAPP, acetoacetyl-CoA, (R)-mevalonate
		PWY-5910	3	0.0212	superpathway of geranylgeranyldiphosphate biosynthesis I (via mevalonate)	acetyl-CoA, DMAPP, acetoacetyl-CoA, (R)-mevalonate

The *p* values indicate the significance of difference in GDM and the healthy controls.

### 2.5. Targeted metabolomics assay

#### 2.5.1. Metabolic extraction

Blood metabolites were extracted from a 100 μL aliquot/subject and transferred to an Eppendorf tube. Subsequently, 300 μL of methanol were added, samples were vortexed for 5 min, and finally centrifugated at 12000 rpm at 4°C for 15 min.

#### 2.5.2. Blood sample analysis by gas chromatography–mass spectrometry

Eighty μL of the supernatant were transferred to an auto-sampler vial for gas chromatography–mass spectrometry (GC–MS) analysis. GC separation was conducted by Agilent 7890B GC System (Agilent Technologies, CA, United States). Mass spectrometry was performed with an EI source with selected ion monitoring using an Agilent 5977A mass spectrometer (Agilent Technologies, CA, United States). Ion source capillary temperature was 230°C.

#### 2.5.3. Ultra-high-performance liquid chromatography–mass spectrometry

The Ultra-high-performance liquid chromatography (UHPLC) analysis was conducted using an Agilent 1,290 Infinity II series UHPLC System (Agilent Technologies, CA, United States). Ion source parameters settings were as follows: capillary voltage = +3,000 V, Nozzle Voltage = +1,500 V, gas (N_2_) temperature = 250°C, gas (N_2_) flow = 11 L/min, sheath gas (N_2_) temperature = 400°C, sheath gas flow = 12 L/min, nebulizer = 35 psi.

For each of the targeted analytes, multiple-reaction monitoring (MRM) parameters were optimized by directly injecting individual analytes’ standard solutions into the mass spectrometer’s API source. Of the two MRM transitions per analyte, the Q1/Q3 showing the highest sensitivity and selectivity were used as the MRM transitions for quantitative monitoring.

Agilent MassHunter Workstation Software (B.10.00, Agilent Technologies) was used for MRM data acquisition and processing.

### 2.6. Hormone level test

Plasma estradiol and progesterone levels were tested by ADVIA Centaur^®^ XP Immunoassay System. Estradiol was measured by double-antibody sandwich chemiluminescene assay. Progesterone was measured by direct competitive chemiluminescene enzyme immunoassay.

### 2.7. Clinical data analysis

To evaluate the association between clinical characteristics and GDM, we performed a hypothesis test for each clinical feature in GDM subjects and controls. If a feature was normally distributed (Shapiro–Wilk test *p* > 0.05), a Student’s t-test was performed. Otherwise, a Wilcoxon test was used. Tests’ *p* values were adjusted using the Benjamini & Hochberg method, and the significance of the difference was determined by an false discovery rate (FDR) of 0.05.

## 3. Results

### 3.1. Clinical characteristics of the study cohort

We established a prospective cohort to investigate the impacts of intestinal microbiota on GDM (Table 1). Of the 54 subjects investigated, 27 were diagnosed with GDM by standard OGTT (GDM subjects), while the remaining 27 were healthy throughout their pregnancy (controls). GDM subjects and controls showed no significant difference in age and BMI. Interestingly, subjects who developed GDM showed a significantly lower weight gain (11.05 ± 2.9 kg) than the controls (13.44 ± 3.88 kg, *p* < 0.05). In early pregnancy, Hemoglobin (HB) levels in the controls (129.11 ± 10.05 g/L) were significantly higher than GDM subjects (123.00 ± 9.64 g/L, *p* < 0.05). Similarly to prior studies (Lain and Catalano, 2007), during the second trimester, GDM subjects manifested significantly higher levels of HbA1c (5.01 ± 0.23%) than the controls (5.41 ± 0.43%, *p* < 0.01).

### 3.2. Changes of gut microbiota in GDM subjects

**Figure 2:**
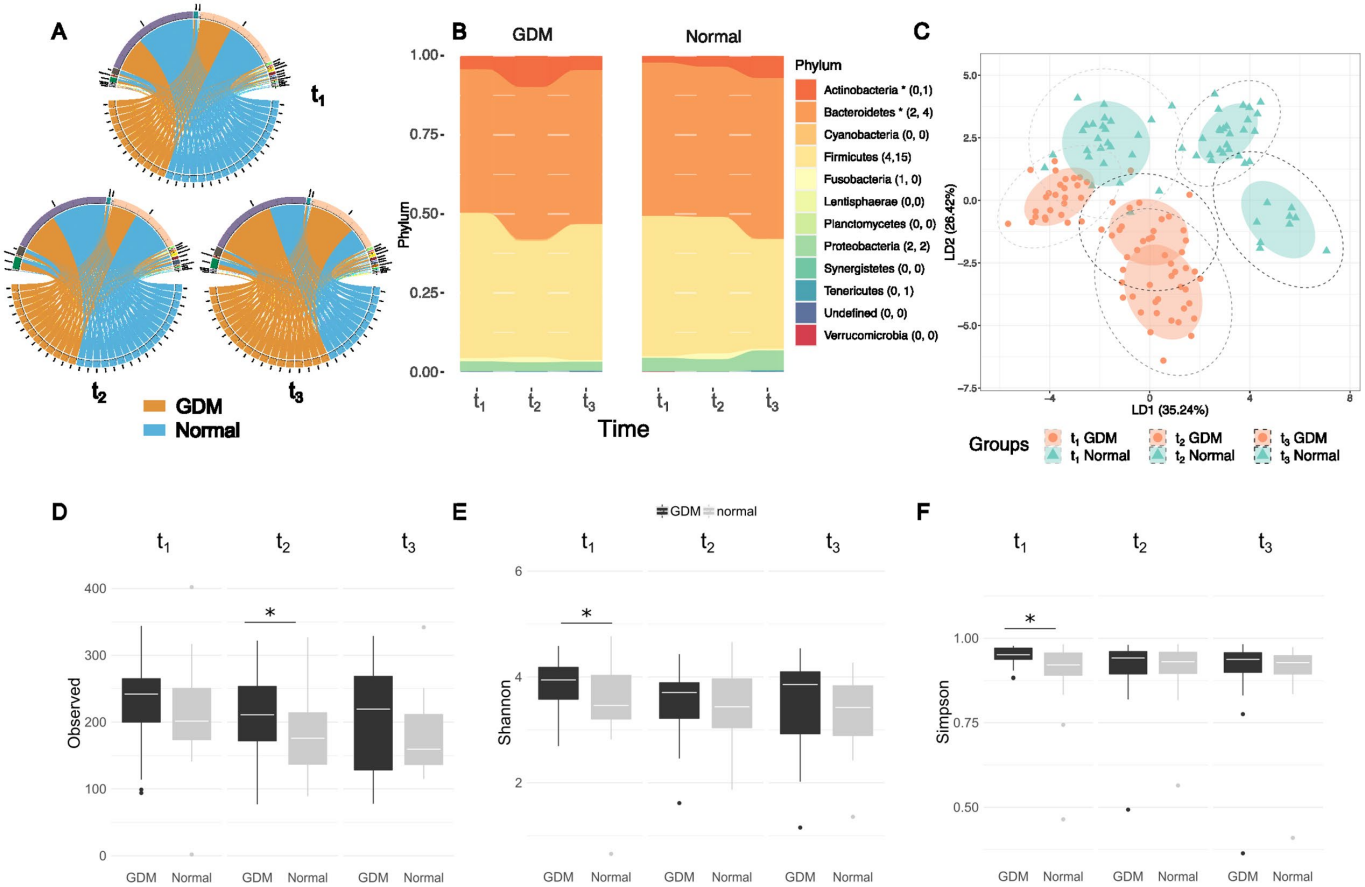
Different abundances and richness of the intestinal microbiota in GDM and healthy controls (labelled as “Normal”) at three time points of pregnancy. **(A)** The top-five most abundant phyla presented at three time points between GDM subjects and healthy controls remain the same although the abundances vary between the two groups. **(B)** The composition ratios of microbial phyla at three time points between GDM subjects and healthy controls, the number of significant Set1- and Set2- OTUs in each phylum is listed in the parentheses. The asteroid indicates overall significance of the phylum. **(C)** Linear discriminant analysis (LDA) based on top-100 variable OTUs in the study cohort show the microbiota vary according to the stage of pregnancy and the onset of GDM. Mean absolute deviation (MAD) is calculated for each OTU to determine the variability. **(D)** Alpha diversity from early pregnancy to postpartum as represented by observed richness, Shannon **(E)** and Simpson indices **(F)** of the microbiome profiles between GDM and healthy controls. Significance was tested at each time points by the Wilcoxon test. “Normal” denotes healthy controls.

The microbial composition of each subject at three time-points is presented in class level (Figure 2A). The top three most abundant phyla were *Firmicutes*, *Bacteriodetes*, and *Actinobacteria* (Supplementary Figure S1), with similar amounts at all three time points, in GDM subjects and controls (Figure 2B; Table 3).

**Table 3:** The five most abundant phyla identified from gut-microbiota of GDM and Normal group in three timepoints of pregnancy.

Phylum	Early pregnancy		Late pregnancy		Postpartum	
	GDM	Control	GDM	Control	GDM	Control
Bacteroidetes	40.40%	43.66%	42.30%	45.76%	48.73%	50.79%
Firmicutes	50.19%	45.34%	44.92%	44.67%	42.95%	34.57%
Actinobacteria	4.95%	2.25%	8.18%	3.74%	4.53%	7.17%
Proteobacteria	3.43%	7.86%	3.09%	3.86%	2.79%	6.20%
Fusobacteria	0.62%	0.68%	1.01%	1.85%	0.51%	0.62%
Total	99.59%	99.79%	99.50%	99.88%	99.51%	99.35%

The abundance of each phylum is based on the total percentage of all OTUs present in that phylum.

We performed linear discriminant analysis (LDA) on the top 100 OTUs with the highest mean absolute deviation (MAD>0) (Figure 2C). Based on the first two LDs (LD1 and LD2), the microbiota was separated according to the time-points and patient grouping (GDM subjects vs. controls). The deviation between GDM subjects and controls was more evident in t_2_ and t_3_, suggesting that the changes in the GDM’s gut microbiota were associated with pregnancy progression. We also conducted Linear discriminant analysis Effect Size (LEfSe) analysis (Segata et al., 2011) for each of the three timepoints to visualize the difference taxa between GDM subjects and healthy control (Supplementary Figure S2).

During pregnancy’s early stages (t_1_), GDM subjects and controls showed no significant difference in gut microbiota diversity (Wilcoxon’s test, *p* > 0.05). However, during the same stage, the Shannon and Simpson index found the microbial community more diverse in the GDM group. In the third trimester (t_2_), GDM subjects showed a significantly higher number of OTUs in gut microbiota than the controls (Wilcoxon’s test, *p* = 0.03, Figures 2D–F). However, according to the Shannon and Simpson index the richness and evenness were not statistically significant. Finally, postpartum (t_3_), alpha-diversity showed no difference between GDM subjects and controls.

### 3.3. Microbial taxa associated with gestational diabetes

We used a permutation test to evaluate the significance of microbial taxa’s changing abundance between two consecutive time points (t_2_ vs. t_1_, t_3_ vs. t_2_) in GDM subjects and controls. Notably, we identified 32 OTUs differently regulated in GDM subjects (FDR < 0.1) (Table 4). Among the 32 OTUs, nine were significantly regulated between t_1_ and t_2_ (Set1-OTUs, Table 4), with different behaviors in GDM subjects vs. controls (Supplementary Figures S4A, S5A). Furthermore, 23 OTUs were significantly regulated in the controls during t_2_ and t_3_ (Set2-OTUs, Table 5). Eleven OTUs in Set2 were suppressed in GDM subjects, while drastically increased in the controls. The other 12 OTUs, increased in the GDM subjects and decreased in the controls (Supplementary Figures S4B, S5B).

**Table 4:** Set1-OTUs with significantly altered abundances between GDM and healthy controls.

Order	Genus	OTU ID	FDR	Abundance: GDM vs. Control
o__Lactobacillales	g__Lactobacillus	f383c2310b0591938662cfc86103cbee	0.000	low vs. increasing
o__Lactobacillales	g__Lactobacillus (s__zeae)	623908ac1fccc394e50027f1dec0d7d5	0.000	increasing vs. decreasing
o__Fusobacteriales	Unassigned	d7e44c932c551da8eeedd8ac53f01a02	0.035	decreasing vs. increasing
o__Caulobacterales	g__Caulobacter	3a98d5ec65c7c79a28b65611e1cc5f3b	0.035	increasing vs. decreasing
o__Bacteroidales	g__Prevotella	dd880d05e7985b728b0292355b402dc4	0.084	low vs. increasing
o__Clostridiales	g__Dialister	73f1eb71de84311115038599adfa4c40	0.093	decreasing vs. increasing
o__Bacteroidales	g__[Prevotella]	adac2e87e83cbfb6d144469e510d6b7c	0.093	low vs. decreasing
o__Burkholderiales	g__Sutterella	dcd619b840bf5c7dc86c231026407b85	0.093	low vs. decreasing
o__Clostridiales	g__Anaerococcus	c0f60859070d06f230c0d077915a74c9	0.093	low vs. increasing

Annotated Set1-OTUs that show significant difference between timepoint t1 and t2. All OTUs are annotated to the most exclusive taxonomic levels.

**Table 5:** Set2-OTUs with significantly altered abundances between GDM and healthy controls.

Order	Genus	OTU ID	FDR	Abundance: GDM vs. Control
o__RF32	Unassigned	1a2237a36f37e9dc6b1d6adaf82ba995	0.000	increasing vs. decreasing
o__Bacteroidales	g__Bacteroides	a8b7c477f24d9eefad61089da9b3737c	0.000	increasing vs. decreasing
o__Burkholderiales	g__Sutterella	dcd619b840bf5c7dc86c231026407b85	0.000	increasing vs. decreasing
o__Clostridiales	g__Faecalibacterium(s__prausnitzii)	f545f1befaa2154503f4d48be98d6f20	0.000	increasing vs. decreasing
o__Clostridiales	g__Faecalibacterium (s__prausnitzii)	47ced4607b5c319fe5021ca041d3d310	0.000	increasing vs. decreasing
o__Clostridiales	g__Peptoniphilus	4f070ff4895d8743d3111d180e25f7ed	0.000	increasing vs. decreasing
o__Bacteroidales	g__Prevotella	0b28c7f9f52a5bc4fa6fc92e3a6a3e61	0.000	low vs. increasing
o__Clostridiales	g__Dorea (s__longicatena)	c5ce48ef1bde6255a8dfc808e4dcd9ba	0.000	increasing vs. decreasing
o__Clostridiales	Unassigned	77c3857f39a914d68117114dae6ff9fa	0.000	increasing vs. decreasing
o__Clostridiales	g__Anaerococcus	6b38e1484786e62b68ddb88194b6d5a7	0.000	increasing vs. decreasing
o__Clostridiales	g__Blautia	1bd9abd8981a87cc18fcab2ea5357043	0.000	increasing vs. decreasing
o__Clostridiales	g__Oscillospira	6db1ecacf0a9069bdc3769d566e6bc14	0.000	increasing vs. decreasing
o__Clostridiales	g__Oscillospira	4513d13bd91f493e23fddc8458ddc242	0.000	increasing vs. decreasing
o__Clostridiales	g__Oscillospira	6ea68b1f9306c6a113f3b54d7dd96980	0.016	stable vs. increasing
o__Coriobacteriales	Unassigned	9634685a7deef3481e6a70c7066abbf7	0.023	decreasing vs. increasing
o__Bacteroidales	g__Bacteroides	583f1c98ae63ff6c82fec113e7935b6a	0.060	low vs. increasing
o__Clostridiales	Unassigned	93e69425555947e705e1a08df6b4314e	0.060	low vs. increasing
o__RF39	Unassigned	529494b7c7acd36d33035ad989f3a6ac	0.060	low vs. increasing
o__Lactobacillales	g__Lactobacillus	ebd417f69f94dd74b6baa60f66202ea8	0.060	low vs. increasing
o__Clostridiales	g__Blautia	2c1587124073059af1de3a13debb8376	0.068	low vs. increasing
o__Bacteroidales	g__Prevotella	ddb902955d5cf7410502b2078b0c19f7	0.070	low vs. increasing
o__Clostridiales	Unassigned	3d6b98c9310d0d9e09e62f9b292f3f41	0.077	low vs. increasing
o__Clostridiales	g__Ruminococcus	176bd59dc7968cb5ac2fbe63c9940e39	0.084	low vs. increasing

Annotated Set2-OTUs that show significant difference between timepoint t_2_ and t_3_. All OTUs are annotated to the most exclusive taxonomic levels. “increasing” and “decreasing” indicate the changing trends of the abundance for each OTU. “low” means the overall abundance of the OTU is very low.

### 3.4. Microbial metabolic pathways influence hormone levels

We predicted the activities of microbial metabolic pathways based on the difference of microbial profiles over two consecutive time points, i.e., t_2_ vs. t_1_ and t_3_ vs. t_2_ (Figure 1). Then, we used a permutation test to evaluate the significance of our findings. Thus, we identified 45 metabolic pathways significantly altered between t_1_ and t_2_ (Set1-pathways, Supplementary Table S2) and 87 between t_2_ and t_3_ (Set2-pathways, Wilcoxon test, *p* < 0.05, Supplementary Table S3). Additionally, we noticed that Set1-pathways showed significantly different activities between GDM subjects and controls (Fisher test *p* < 0.05, Supplementary Figure S3A).

**Figure 3:**
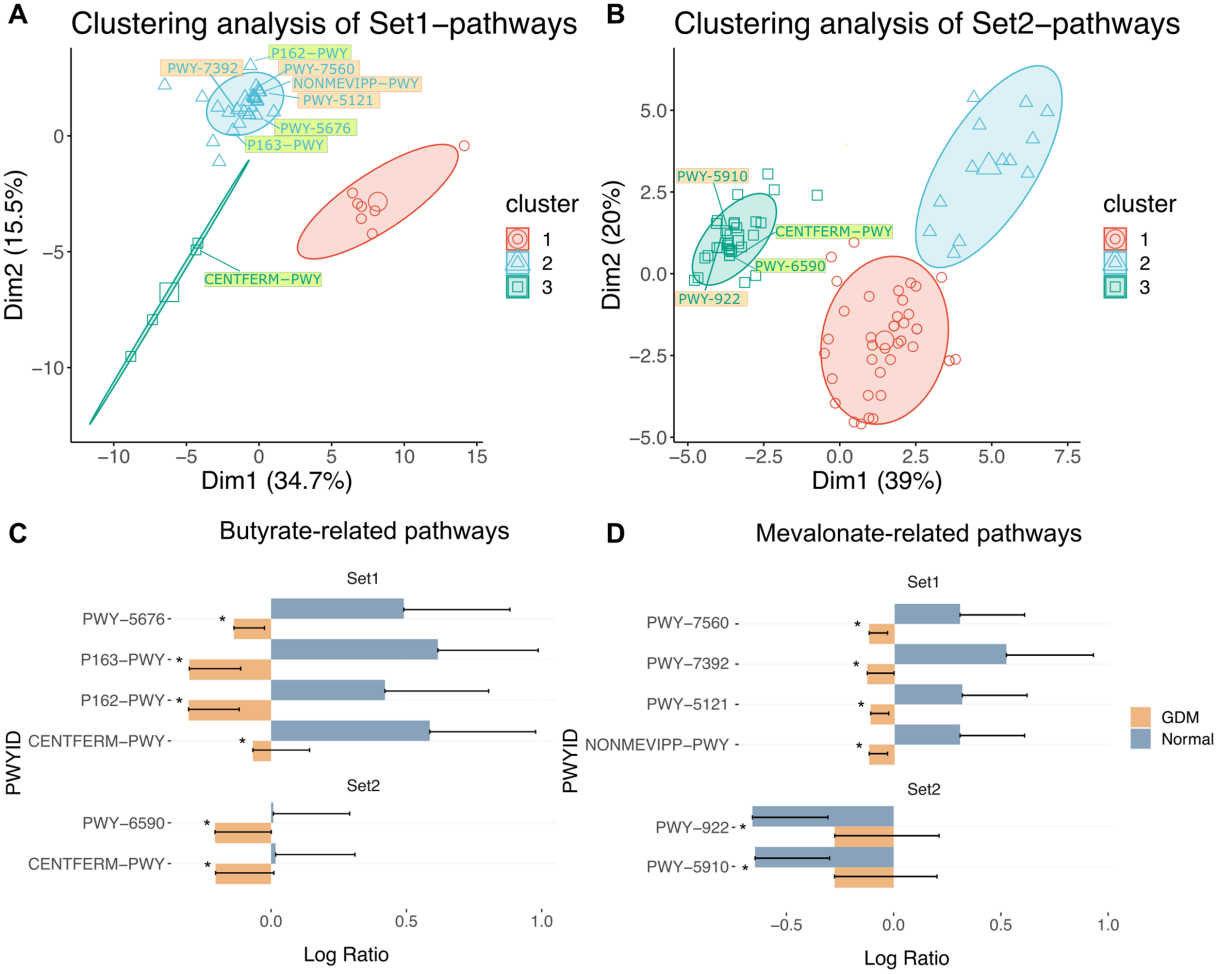
Clustering of inferred significantly altered microbial metabolic pathways from Set1-OTUs and Set2-OTUs. PCA of significantly altered microbial metabolic pathways corresponding to Set1-OTUs **(A)** and Set2-OTUs **(B)** by permutation test. Clustering of pathways by k-means algorithm are labeled by colored circles. Pathway IDs highlighted in light green are butyrate-related pathways, those highlighted in light orange are mevalonate-related pathways. Pathways in Table 5 were labelled in each cluster. **(C)** butyrate-related and pathways in Set1 and Set2: PWY-5676 (acetyl-CoA fermentation to butanoate II), P163-PWY (L-lysine fermentation to acetate and butanoate), P162-PWY (L-glutamate degradation V (via hydroxyglutarate)), CENTFERM-PWY (pyruvate fermentation to butanoate), PWY-6590 (superpathway of *Clostridium acetobutylicum* acidogenic fermentation). **(D)** mevalonate-related pathways in Set1 and Set2: PWY-7560 (methylerythritol phosphate pathway II), PWY-7392 (Engineered Pathway: taxadiene biosynthesis (engineered)), PWY-5121 (superpathway of geranylgeranyl diphosphate biosynthesis II (via MEP)), NONMEVIPP-PWY (methylerythritol phosphate pathway I), PWY-922 (mevalonate pathway I (eukaryotes and bacteria)), PWY-5910 (superpathway of geranylgeranyldiphosphate biosynthesis I (via mevalonate)). Significance of alteration were tested by permutation test (^***^ *p* < 0.001, ^**^ *p* < 0.01, ^*^ *p* < 0.05). “Normal” denotes healthy controls.

Then we performed the principal component analysis (PCA) based on the significant metabolic pathways’ activities followed by K-means clustering (Figures 3A,B). We observed that the Set1-pathways (Figure 3A; Supplementary Table S2; Supplementary Figure S6) were clustered into three distinct groups. The first contained seven pathways, 6 of which were biosynthetic. The second contained 34 pathways, mainly for nucleotide biosynthesis, cell structure, and degradation pathways. Finally, the third group contained four pathways, mostly related to energy metabolism.

Similarly, 87 Set2-pathways (*p* < 0.05) formed three groups (Figure 3B; Supplementary Figure S7; Supplementary Table S3). The first contained 39 pathways, predominantly biosynthesis and degradation processes of amino acids and aromatic compounds. The second group contained 13 pathways of aromatic compounds degradation. Finally, the last group contained 35 pathways, mostly biosynthetic.

To identify the critical metabolites in the microbial metabolic pathways associated with GDM, we retrieved from the MetaCyc database (Caspi et al., 2020) all the metabolites for each group of significant pathways. Then, we ranked the metabolites based on recurrence frequency per group (Supplementary Table S4). In Set1-pathways, we noticed that butyrate-related (*n* = 20) and mevalonate-related metabolites (*n* = 16) were both highly represented (Supplementary Table S4). Finally, we identified similar metabolites in the Set2-pathways but with a moderate frequency. We then retrieved 8 Set1-pathways and 4 Set2-pathways in which butyrate and mevalonate metabolites were overrepresented (Figures 3C,D; Supplementary Figures S8A,B; Table 2).

Overall, our data show a difference in the gut microbiota of GDM subjects vs. controls, leading to a drastic change of microbial metabolic pathways, potentially linked with GDM’s onset.

### 3.5. Validation of the blood metabolites and hormone levels in GDM

**Figure 4:**
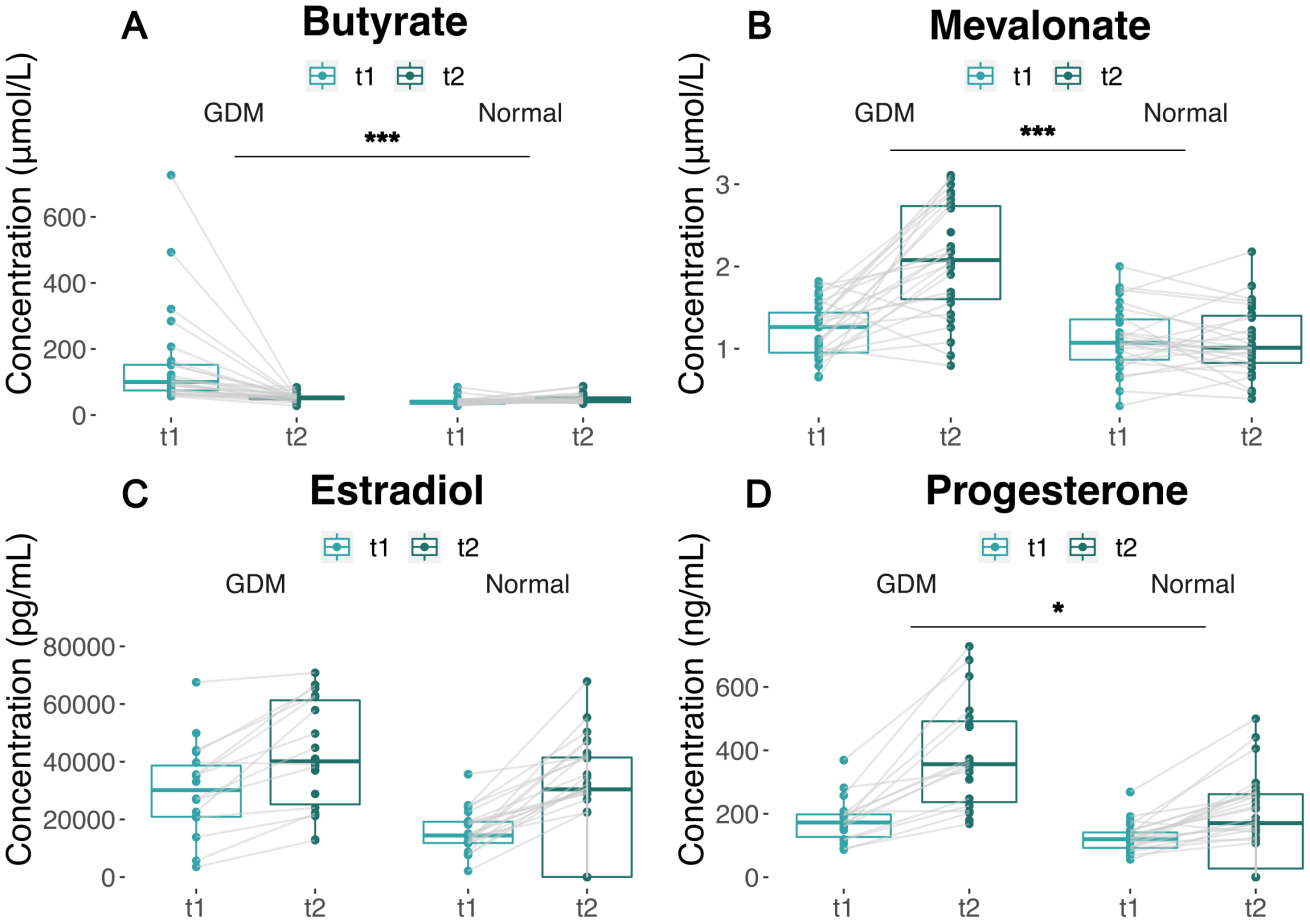
Covariation of metabolites and hormone levels in GDM subjects and controls between t_1_ and t_2_. **(A,B)** Plasma butyrate and mevalonate levels of GDM and normal group between t_1_ and t_2_. Measures from the same patient are linked by solid lines. **(C,D)** Estradiol and progesterone levels of GDM and normal group between t_1_ and t_2_. Measures from the same patient are linked by solid lines. The significance of the difference between GDM subjects and normal group are based on Wilcoxon’s rank-sum test *p* values (^***^ *p* < 0.001, ^**^ *p* < 0.01, ^*^ *p* < 0.05). “Normal” denotes healthy controls.

To verify whether blood butyrate and mevalonate levels elevate with the increasing activities of the corresponding microbial pathways, we performed a targeted metabolomics assay using the blood samples of an independent cohort (28 GDM subjects and 28 controls) at t_1_ and t_2_, respectively. As a result, butyrate levels in GDM subjects were higher than the controls (*p* < 0.001) at t_1_, while they decreased to similarly low levels during late pregnancy (Figure 4A; Supplementary Figure S9A). Mevalonate levels remained similar in GDM subjects and controls at t_1_; but increased significantly in GDM subjects at t_2_ (*p* < 0.001, Figure 4B; Supplementary Figure S9B).

In the same cohort, we also measured estradiol and progesterone levels, which increased with the length of pregnancy from t_1_ to t_2_ (Figures 4C,D), in both groups_._ However, GDM subjects showed higher estradiol levels at t_1_ (Wilcoxon’s test *p* < 0.005, Supplementary Figure S9C), and higher progesterone levels at both t_1_ and t_2_ (Wilcoxon’s test *p* < 0.001) (Supplementary Figure S9D). The increase in estradiol and progesterone levels in GDM was consistent to the changes of butyrate and mevalonate levels, which are coupled with the suppression of butanoate and Isopentenyl Diphosphate Biosynthesis (Supplementary Figure S10). Of note, our data show Isopentenyl Diphosphate Biosynthesis tend to increase in the normal controls (PWY-592, PWY-5910) but remain unchanged or decrease in GDM subject, which contributes to the accumulation of mevalonate.

## 4. Discussion

GDM is a common metabolic disorder during pregnancy, known to be associated with changes in the gut microbiome. However, to date, microbiota’s possible mechanisms and relevant metabolites in GDM subjects remain unclear. In this study, we identified a set of bacterial taxa associated with GDM’s onset in early and late pregnancy, and postpartum. We found that GDM-associated taxa are enriched in butanoate and mevalonate metabolic pathways in GDM subjects, resulting in increased levels of pregnancy hormones, further contributing to insulin resistance.

Although the interaction between microbiota and GDM is widely reported, most of the current knowledge is based on cross-sectional design, making it challenging to establish causal relationships. The major advantage of the current study is based on self-controlled, time-dependent profiling of gut microbiota in GDM subjects and their healthy counterparts, via which we are able to obtain highly relevant changes in microbiota involved in the onset of GDM. This approach is more specific than cross-sectional design and augmented by stringent process for fecal specimen collection, preserving microbiota’s richness and enhancing the accuracy of the analysis. Our findings suggest that variations in gut microbiota contribute to the pathogenesis of GDM through individual hormonal metabolism, as represented by specific metabolites of pregnancy hormones. These results help differentiate the causal effects of microbiota on hormonal changes in GDM from other conflicting reports.

We report two sets of microbial taxa, significantly associated with GDM in the second and third trimester. In Set1-OTUs, *Veillonellaceae* was more abundant in GDM, and reported as a potential target for diabetes and hyperlipidemia management (Liu et al., 2019), and as a marker of euglycemia during pregnancy (Chen et al., 2021). As previously published, *Fusobacteriaceae* was suppressed in GDM subjects between t_1_ and t_2_, whereas the controls showed an opposite tendency (Gomez-Arango et al., 2016). In Set2-OTUs, *Dorea* (Family *Lachnospiraceae*) was reported to be associated with type II diabetes (Li et al., 2020) and GDM (Ferrocino et al., 2018). Furthermore, our results showed that *Prevotellaceae* (Clemente et al., 2015; Martínez et al., 2015) was depleted in GDM subjects, confirming a recent study (Chen et al., 2021). Moreover, we reported for the first time some microbial taxa in association with GDM, such as *Caulobacteraceae*. Although little is known of the relationship between gut microbiota and pregnancy hormones, a previous study showed that the interruption of microbiota, because of antibiotic treatment for example, indeed impacts the host’s metabolic pathways, including steroid hormones synthesis (Antunes et al., 2011).

The relationship between gut microbes and hormones such as progesterone and estradiol levels has been reported in previous studies in healthy and GDM subjects (Nuriel-Ohayon et al., 2019; Rold et al., 2022). During pregnancy, certain microbes, such as *Bifidobacterium* in microbiota interacts with hormones, including progesterone, estrogen and insulin, and plays a significant role in reproductive endocrinological processes (Nuriel-Ohayon et al., 2019; Qi et al., 2021). In GDM subjects, a high-fat and low-fiber diet, along with altered gut microbiota, has been shown to contribute to abnormal glucose metabolism, insulin resistance, and increased risk of GDM incidence (Hasain et al., 2020). While cumulative evidence has shown an association between hormonal changes and changes in the microbiota, none of the included studies compared the hormonal change between GDM and non-GDM women in light of time-varying microbiota during pregancy. Therefore, the implication of the covariation between hormones on the gut microbiota in GDM remains unclear (Rold et al., 2022). Our study addresses the gap in the current literature by comparison of temporal variation of serum estradiol and progesterone levels between women with GDM and healthy controls, which provides insight into the potential role of the interaction of gut microbiota and hormone metabolism in GDM.

Metabolic pathways differ between healthy pregnant women and those with GDM, for example, arginine metabolic pathway, beta-oxidation, urea cycle pathway, which also resemble type 2 diabetes (DM2) (Spanou et al., 2022). Specific metabolites were primarily involved in the metabolic disorders of GDM, such as 3-methyl-2-oxovaleric acid, branch-chain amino acids, isobutyric acid (Lu et al., 2021). Nevertheless, there is a lack of consistency among the altered metabolic pathways across different studies.

Our data suggests that microbiota changes in GDM are likely to affect the butyrate and mevalonate metabolic pathways. Butanoate pathways are known to influence insulin sensitivity by increased production of butyrate (Gao et al., 2009). In our data, both butyrate-related pathways’ activities and butyrate levels showed a drastic decrease from significantly high levels at t_1_ in GDM subjects vs. controls, where butyrate levels remain stably low. Additionally, we report a set of significantly altered OTUs contributing to these pathways (Figures 3C,D), all previously reported as butyrate-producers (Bäckhed et al., 2015; Lopez-Siles et al., 2017; Wu et al., 2018; Ozato et al., 2019). Interestingly, mevalonate metabolic pathways were less known for their effects on GDM until a recent review mentioned that the levels of a mevalonate-related metabolite, isopentenyl phosphate (IPP), were significantly altered in GDM subjects (Roverso et al., 2021). Several mevalonate-related metabolites found in our study, including IPP, DMAPP, and GGPP, are precursors for steroid hormones. These hormones are involved in metabolic reprogramming and increased GDM risk (Holstein and Hohl, 2004). Notably, we detected significantly higher levels of mevalonate in GDM subjects at t_2_ with consistent increase of estradiol and progesterone levels. Our findings indicate that metabolites of microbial origin can contribute to the aberrant levels of steroid hormones in association with insulin resistance in GDM.

Nevertheless, the current study has several limitations. First, a small sample size. Second, the 16S rRNA gene-based taxonomy annotation is less sensitive than shot-gun metagenome, failing to annotate several OTUs to species level. Third, while our study identified butyrate and mevalonate as intermediary metabolites of gut microbiota in GDM, their enrichment significance was not directly verified, and other intermediary metabolites are yet to be discovered. Finally, our validation confirmed that estradiol and progesterone changes were inconsistent in GDM subjects. Many other pregnancy hormones are also associated with insulin resistance. However, as the hormone levels in pregnant women vary drastically due to physiological changes, the current study is not sensitive enough to detect all relevant hormonal changes in GDM in response to microbiota.

In this study, we generated self-controlled, temporal profiles of microbiota for GDM subjects and their healthy counterparts using 16S rRNA gene sequencing and identified critical pathways with overrepresented metabolites, which were further validated in targeted metabolomics assay. Our findings shed light on a novel perspective regarding the elusive pathogenesis of GDM and contributed to expanding our comprehension of the correlation between microbiota and the onset of GDM. Our results revealed important insights into the interaction mechanisms among hormonal changes, gut microbiome alterations, and metabolites in the context of GDM.

## Data availability statement

The datasets presented in this study can be found in online repositories. The name of the repository and accession number can be found at: NCBI; PRJNA963229.

## Ethics statement

The studies involving human participants were reviewed and approved by the Ethics Committee of the First Affiliated Hospital of Xiamen University (reference number KY2022-033). The patients/participants provided their written informed consent to participate in this study.

## Author contributions

QL and QC conceived the study. SW, XL, LC, and YiZ (6th author) collected all clinical information and specimens. SW, LC, and YaZ conducted sample preparation. SW, XL, LC, and YaZ conducted the experiments. XL and JZ analyzed the data. QL, QC, XL, and YiZ (7th author) wrote the manuscript. All authors have given the consent for publishing the manuscript. All authors contributed to the article and approved the submitted version.

## Funding

This research was supported by the National Natural Science Foundation of China (No. 82272944), the Natural Science Foundation 1145 of Fujian Province (No. 2021J05298) and Xiamen Medical and Health 1146 Science and Technology Project (No. 3502Z20194011).

## Conflict of interest

The authors declare that the research was conducted in the absence of any commercial or financial relationships that could be construed as a potential conflict of interest.

## Publisher’s note

All claims expressed in this article are solely those of the authors and do not necessarily represent those of their affiliated organizations, or those of the publisher, the editors and the reviewers. Any product that may be evaluated in this article, or claim that may be made by its manufacturer, is not guaranteed or endorsed by the publisher.

## Footnotes

^1^ https://github.com/picrust/picrust2
