# Supplementary material for: Gut microbiome interacts with pregnancy hormone metabolites in gestational diabetes mellitus
Source: Front Microbiol. 2023 Jul 10;14:1175065. doi: 10.3389/fmicb.2023.1175065 (PMC10364628; doi:10.3389/fmicb.2023.1175065)
Supplement: Supplementary file 1 [file Data_Sheet_1.zip › Supplementary Figure Captions.DOCX]

Supplementary Material

**Supplementary figure caption**

**Figure S1**

Abundance of the main phyla in the study cohort.

**Figure** **S2**

Differentially abundant taxa of gut microbiota in GDM subjects and healthy controls. Cladogram of linear discriminant analysis effect size (LEfSe) analysis displaying phylogenetic distribution of gut microbiota between the GDM subjects and healthy controls in (A) t_1_, (B) t_2_, and (C) t_3_. LDA scores (log10) > 2 and P < 0.05 (A and B) or 0.1 (C) are shown. Bar plots indicating significant bacterial differences between the GDM subjects and healthy in (D) t_1_, (E) t_2_, and (F) t_3_.

**Figure S3**

Inferred significantly altered microbial metabolic pathways from Set1-OTUs and Set2-OTUs. (A) heatmap of Set1-pathways with hierarchical clustering (above) show significant association with onset of GDM by Fisher exact test (P=0.0176). (B) heatmap of Set2-pathways with hierarchical clustering (above) show no significance by Fisher exact test (P>0.05).

**Figure S4**

Line charts of operational taxonomic units which are significantly differently altered in GDM patients. (A) OTUs significantly differently altered between t_2_ and t_1_ (Set1-OTUs) and (B) OTUs significantly differently altered between t_3_ and t_2_ (Set2-OTUs). All OTUs were annotated in phylum level. Significance of alteration were tested by permutation test (*** P<0.001, ** P<0.01, * P<0.05).

**Figure S5**

Operational taxonomic units which are significantly differently altered in GDM patients. (A) OTUs significantly differently altered between t_2_ and t_1_ (Set1-OTUs) and (B) OTUs significantly differently altered between t_3_ and t_2_ (Set2-OTUs). All OTUs were annotated in phylum level. Significance of alteration were tested by permutation test (*** P<0.001, ** P<0.01, * P<0.05).

**Figure S6**

Activities of inferred significantly altered microbial metabolic pathways from Set1-OTUs, or Set1-pathways (n=45) at t_1_, t_2_, and t_3_.

**Figure S7**

Activities of inferred significantly altered microbial metabolic pathways from Set2-OTUs, or Set2-pathways (n=87) at t_1_, t_2_, and t_3_.

**Figure S8**

Line charts of (A) butyrate-related and (B) mevalonate-related pathways. Significance of alteration is based on permutation test (*** P<0.001, ** P<0.01, * P<0.05).

**Figure S9**

Metabolites and hormone levels of GDM patients and normal group at t_1_, t_2_ and t_3_. (A) and (B) Plasm butyrate and mevalonate levels of GDM and normal group at t_1_, t_2_ and t_3_. Comparison is conducted at each timepoint. Black dots represent GDM patients, and grey points represent normal subjects. (C) and (D) Estradiol and progesterone levels of GDM and normal group at t_1_, t_2_ and t_3_. Comparison is conducted at each timepoint.
