# Supplementary material for: Gut microbiome interacts with pregnancy hormone metabolites in gestational diabetes mellitus
Source: Front Microbiol. 2023 Jul 10;14:1175065. doi: 10.3389/fmicb.2023.1175065 (PMC10364628; doi:10.3389/fmicb.2023.1175065)

# Supplementary

**Figure S1**

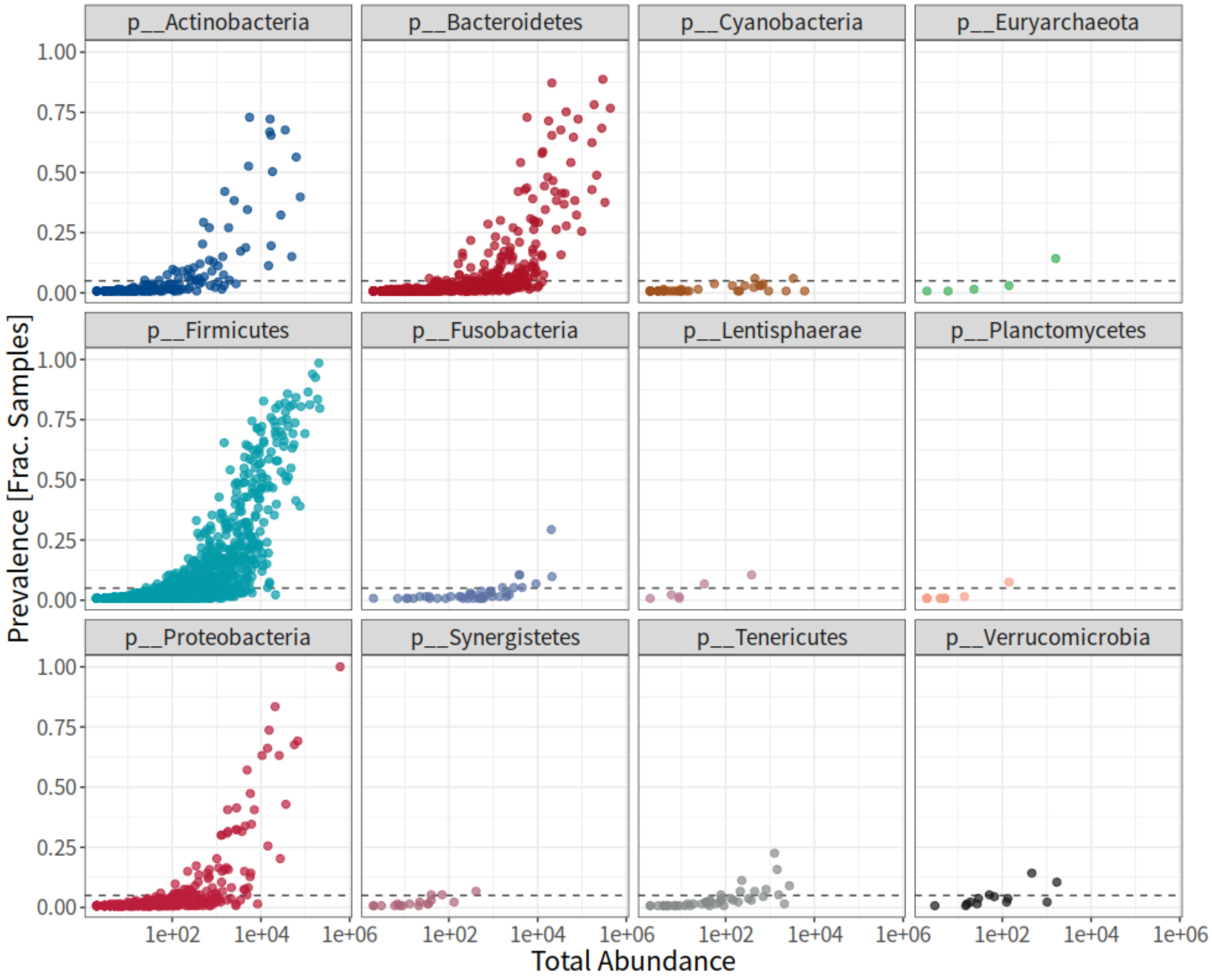

p__Actinobacteria
p__Bacteroidetes
p__Cyanobacteria
p__Euryarchaeota
p__Firmicutes
p__Fusobacteria
p__Lentisphaerae
p__Planctomycetes
p__Proteobacteria
p__Synergistetes
p__Tenericutes
p__Verrucomicrobia
Prevalence [Frac. Samples]
1.00
0.75
0.50
0.25
0.00
1e+02
1e+04
1e+06
Total Abundance

# Figure S2

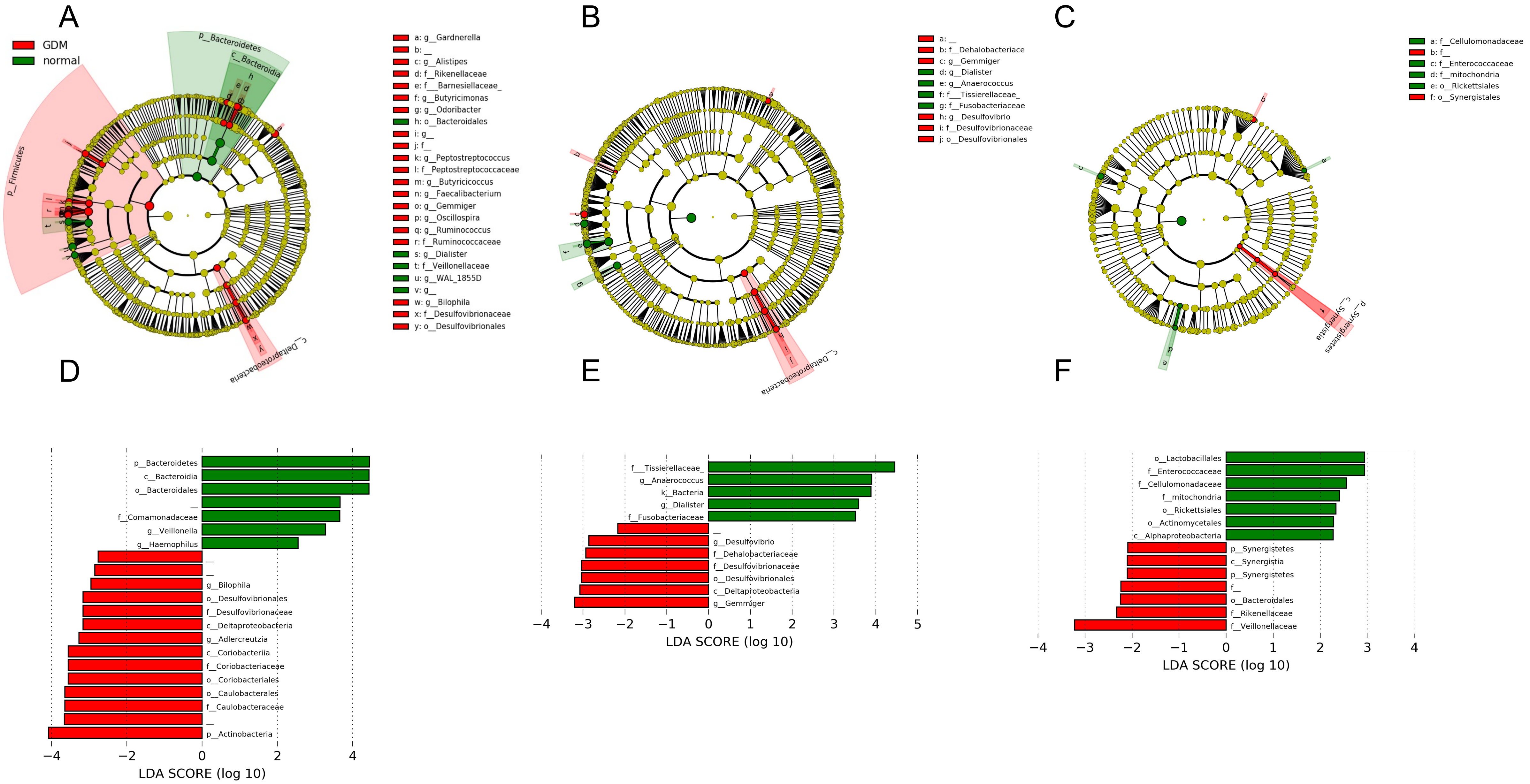
A
B
C
D
E
F
GDM
normal
p__Bacteroidetes
c__Bacteroidia
p__Firmicutes
c__Deltaproteobacteria
a: g__Gardnerella
b: __
c: g__Alistipes
d: f__Rikenellaceae
e: f__Barnesiellaceae_
f: g__Butyricimonas
g: g__Odoribacter
h: o__Bacteroidales
i: g__
j: f__
k: g__Peptostreptococcus
l: f__Peptostreptococcaceae
m: g__Butyricicoccus
n: g__Faecalibacterium
o: g__Gemmiger
p: g__Oscillospira
q: g__Ruminococcus
r: f__Ruminococcaceae
s: g__Dialister
t: f__Veillonellaceae
u: g__WAL_1855D
v: g__
w: g__Bilophila
x: f__Desulfovibrionaceae
y: o__Desulfovibrionales
a: __
b: f__Dehalobacteriace
c: g__Gemmiger
d: g__Dialister
e: g__Anaerococcus
f: f__Tissierellaceae_
g: f__Fusobacteriaceae
h: g__Desulfovibrio
i: f__Desulfovibrionaceae
j: o__Desulfovibrionales
a: f__Cellulomonadaceae
b: f__
c: f__Enterococcaceae
d: f__mitochondria
e: o__Rickettsiales
f: o__Synergistales
c__Synergistia
p__Synergistetes
p__Bacteroidetes
c__Bacteroidia
o__Bacteroidales
__
f__Comamonadaceae
g__Veillonella
g__Haemophilus
__
__
g__Bilophila
o__Desulfovibrionales
f__Desulfovibrionaceae
c__Deltaproteobacteria
g__Adlercreutzia
c__Coriobacteriia
f__Coriobacteriaceae
o__Coriobacteriales
o__Caulobacterales
f__Caulobacteraceae
__
p__Actinobacteria
−4 −2 0 2 4
LDA SCORE (log 10)
f__Tissierellaceae_
g__Anaerococcus
k__Bacteria
g__Dialister
f__Fusobacteriaceae
__
g__Desulfovibrio
f__Dehalobacteriaceae
f__Desulfovibrionaceae
o__Desulfovibrionales
c__Deltaproteobacteria
g__Gemmiger
−4 −3 −2 −1 0 1 2 3 4 5
LDA SCORE (log 10)
o__Lactobacillales
f__Enterococcaceae
f__Cellulomonadaceae
f__mitochondria
o__Rickettsiales
o__Actinomycetales
c__Alphaproteobacteria
p__Synergistetes
c__Synergistia
p__Synergistetes
f__
o__Bacteroidales
f__Rikenellaceae
f__Veillonellaceae
−4 −3 −2 −1 0 1 2 3 4
LDA SCORE (log 10)

# Figure S3

A

Set1-pathways

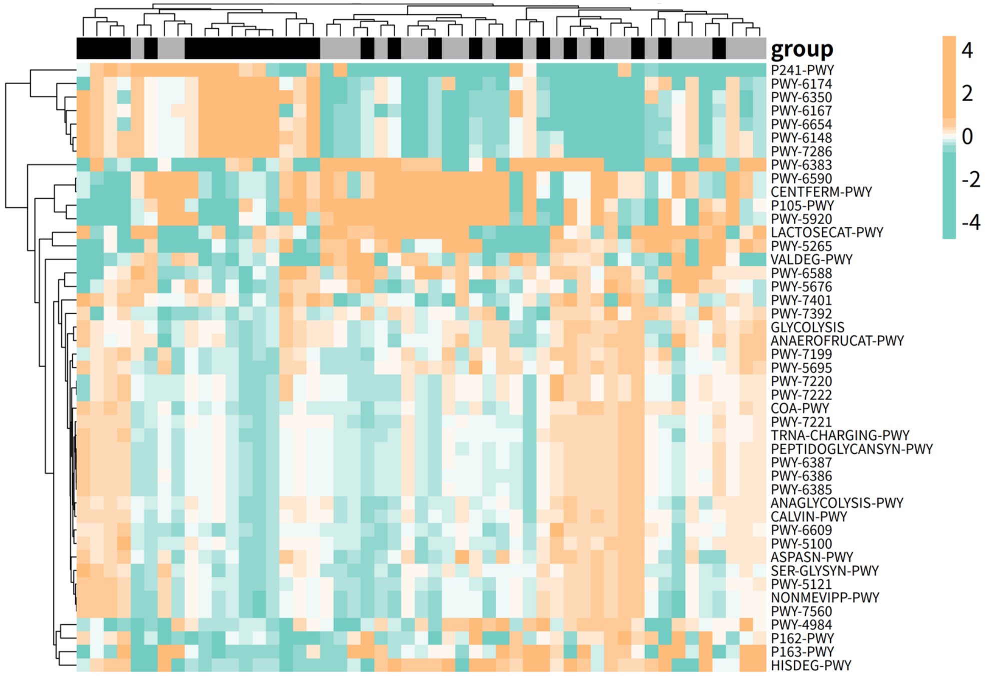


| Fisher exact test | | |
|---|---|---|
| | Normal | GDM |
| cluster1 | 20 | 13 |
| cluster2 | 4 | 14 |

p-value = 0.0176
OR = 5.20

B

Set2-pathways

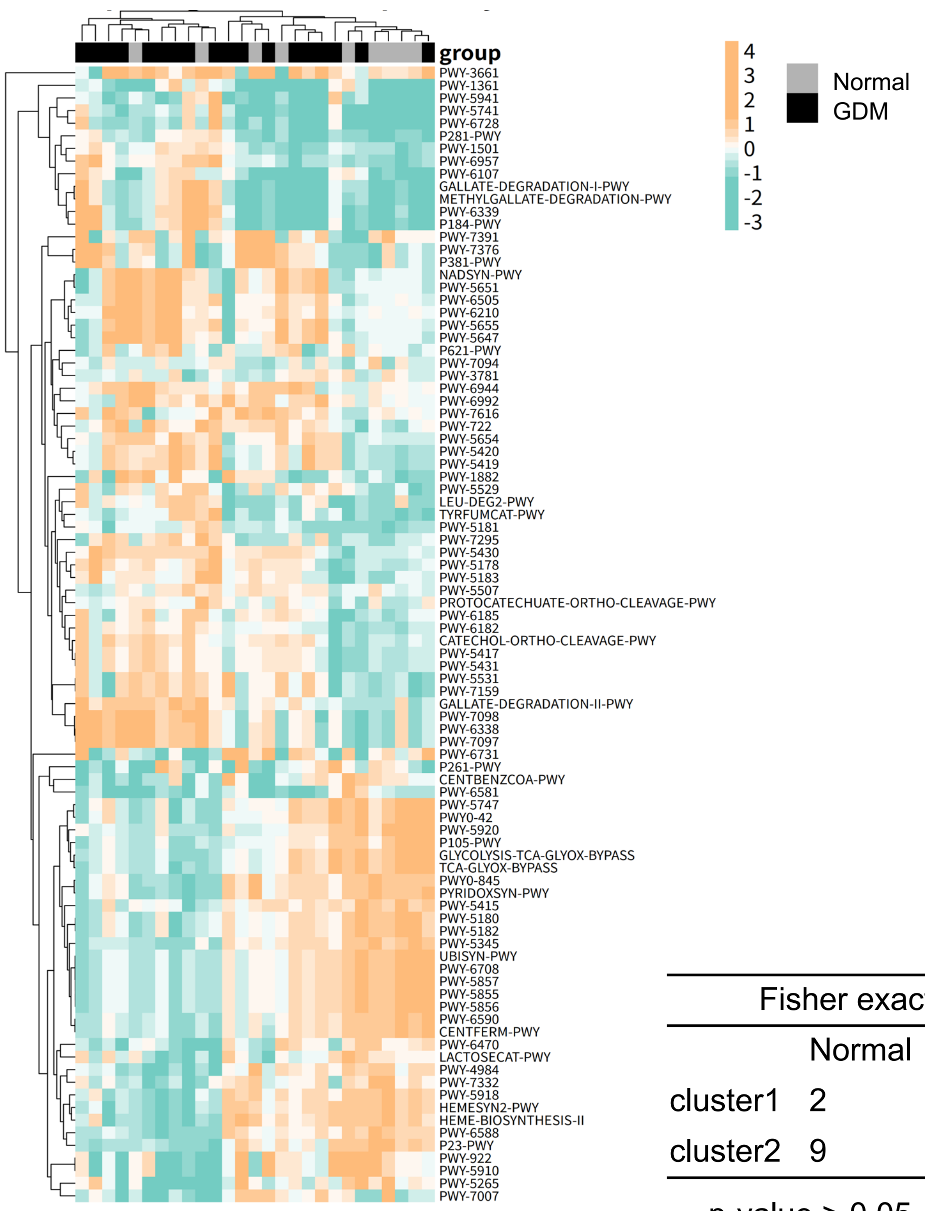


| Fisher exact test | | |
|---|---|---|
| | Normal | GDM |
| cluster1 | 2 | 7 |
| cluster2 | 9 | 19 |

p-value > 0.05

**Figure S4**

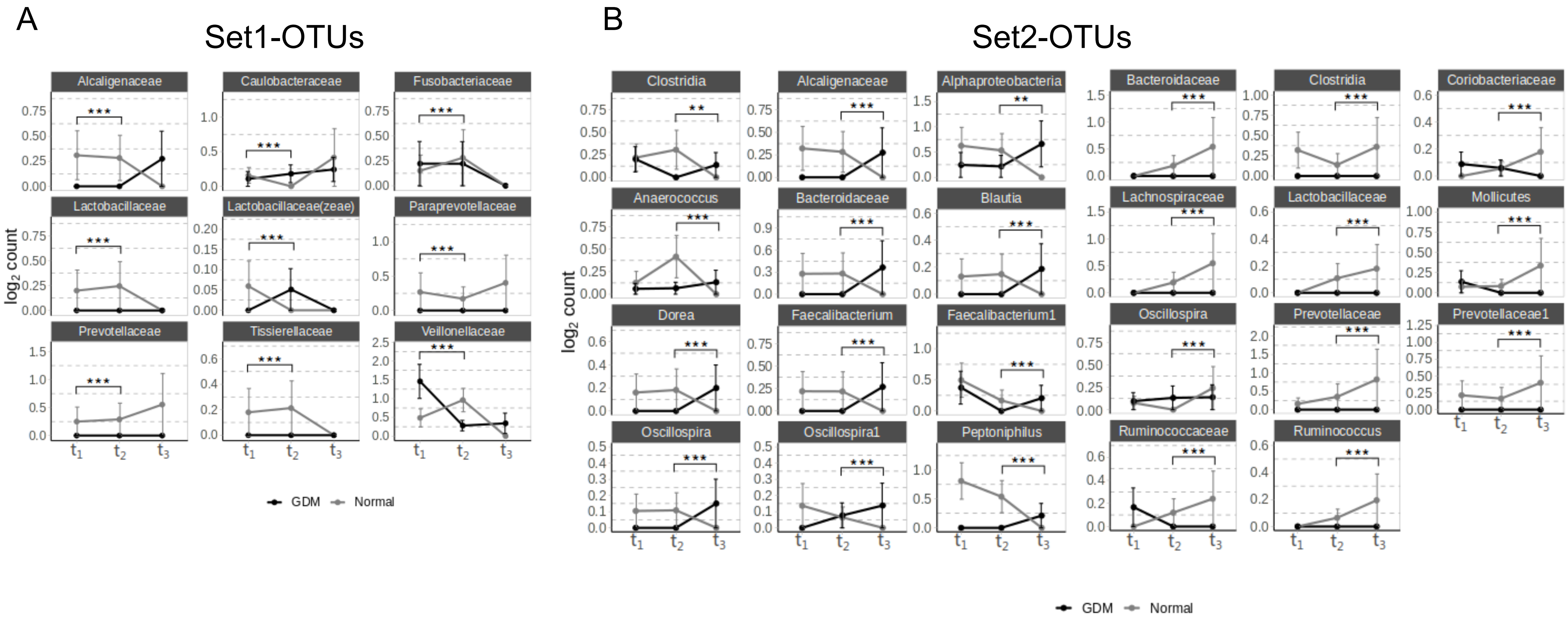
A
Set1-OTUs
Alcaligenaceae
Caulobacteraceae
Fusobacteriaceae
Lactobacillaceae
Lactobacillaceae(zeae)
Paraprevotellaceae
Prevotellaceae
Tissierellaceae
Veillonellaceae
log2 count
GDM
Normal
B
Set2-OTUs
Clostridia
Alcaligenaceae
Alphaproteobacteria
Bacteroidaceae
Clostridia
Coriobacteriaceae
Anaerococcus
Bacteroidaceae
Blautia
Lachnospiraceae
Lactobacillaceae
Mollicutes
Dorea
Faecalibacterium
Faecalibacterium1
Oscillospira
Prevotellaceae
Prevotellaceae1
Oscillospira
Oscillospira1
Peptoniphilus
Ruminococcaceae
Ruminococcus
log2 count
GDM
Normal

# Figure S5

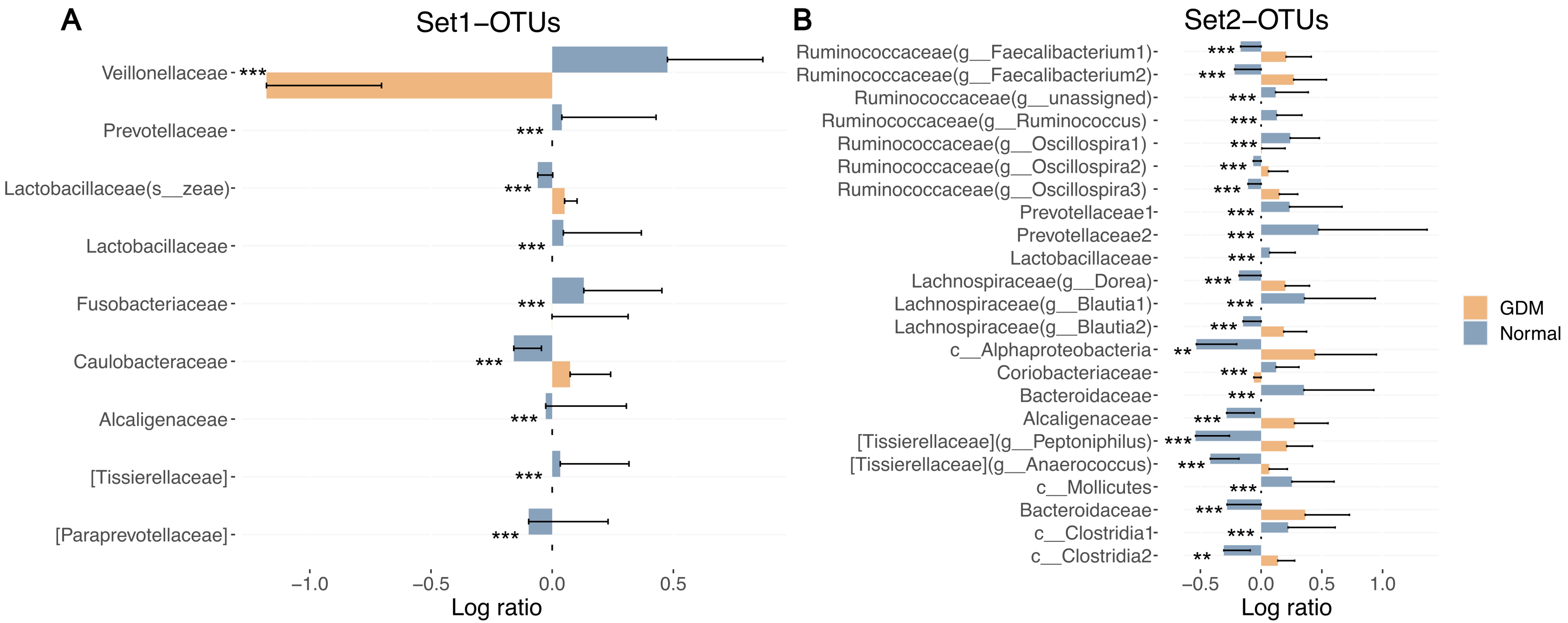
A
Set1−OTUs
Veillonellaceae
Prevotellaceae
Lactobacillaceae(s__zeae)
Lactobacillaceae
Fusobacteriaceae
Caulobacteraceae
Alcaligenaceae
[Tissierellaceae]
[Paraprevotellaceae]
***
−1.0
−0.5
0.0
0.5
Log ratio
B
Set2−OTUs
Ruminococcaceae(g__Faecalibacterium1)
Ruminococcaceae(g__Faecalibacterium2)
Ruminococcaceae(g__unassigned)
Ruminococcaceae(g__Ruminococcus)
Ruminococcaceae(g__Oscillospira1)
Ruminococcaceae(g__Oscillospira2)
Ruminococcaceae(g__Oscillospira3)
Prevotellaceae1
Prevotellaceae2
Lactobacillaceae
Lachnospiraceae(g__Dorea)
Lachnospiraceae(g__Blautia1)
Lachnospiraceae(g__Blautia2)
c__Alphaproteobacteria
Coriobacteriaceae
Bacteroidaceae
Alcaligenaceae
[Tissierellaceae](g__Peptoniphilus)
[Tissierellaceae](g__Anaerococcus)
c__Mollicutes
Bacteroidaceae
c__Clostridia1
c__Clostridia2
***
**
−0.5
0.0
0.5
1.0
Log ratio
GDM
Normal

**Figure S6**

# Set1-pathways

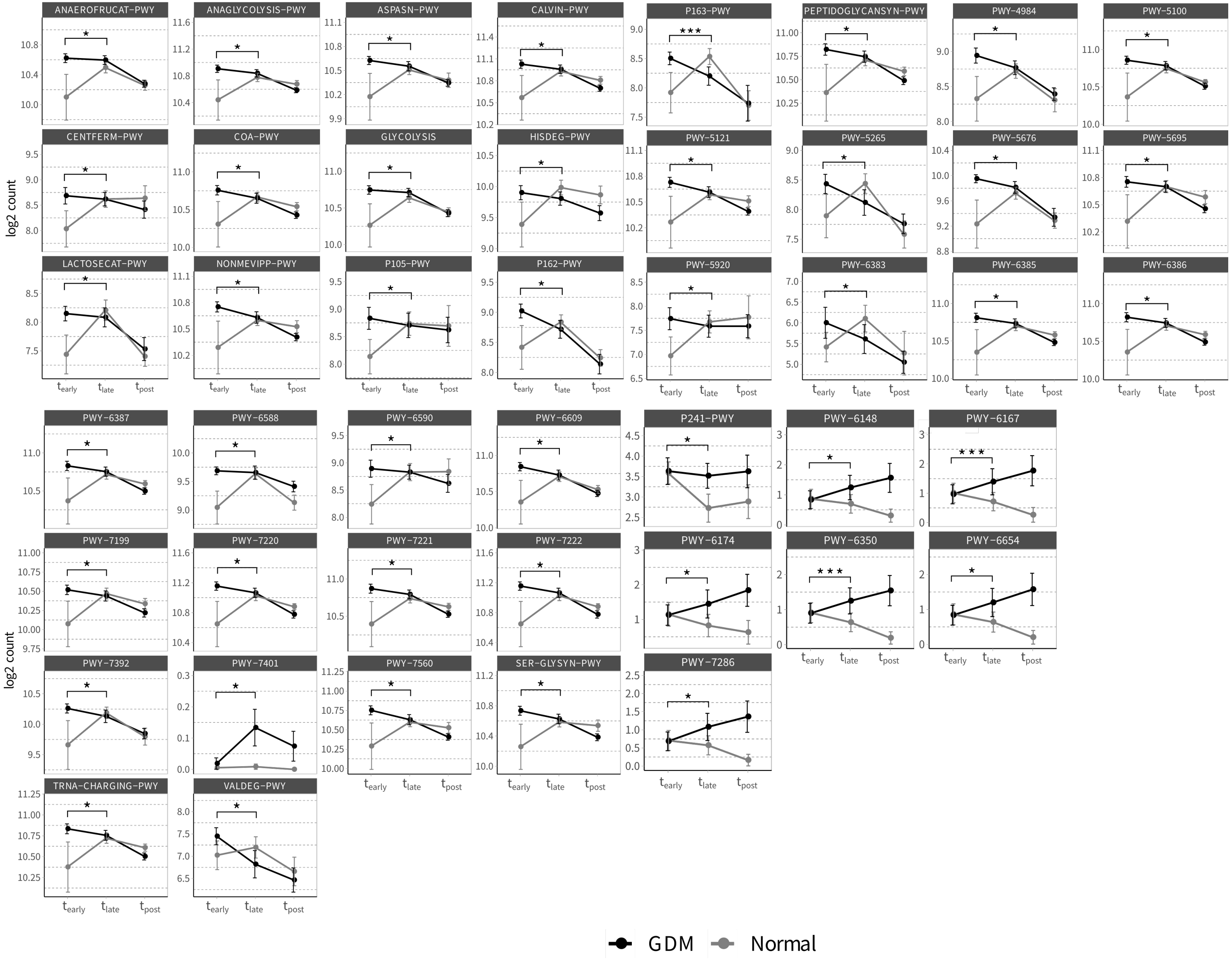

**Figure S7**

# Set2-pathways

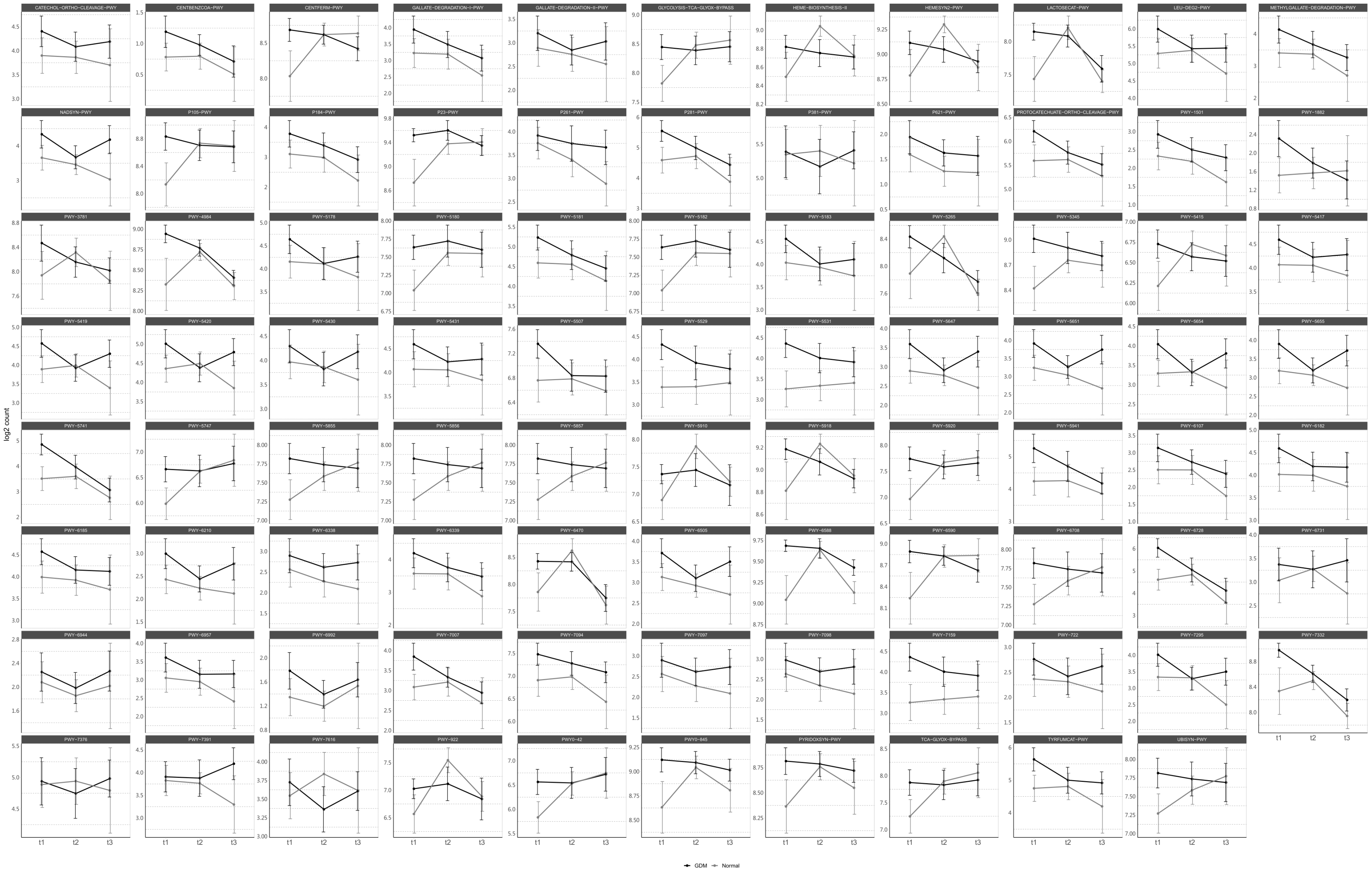

# Figure S8

A

B

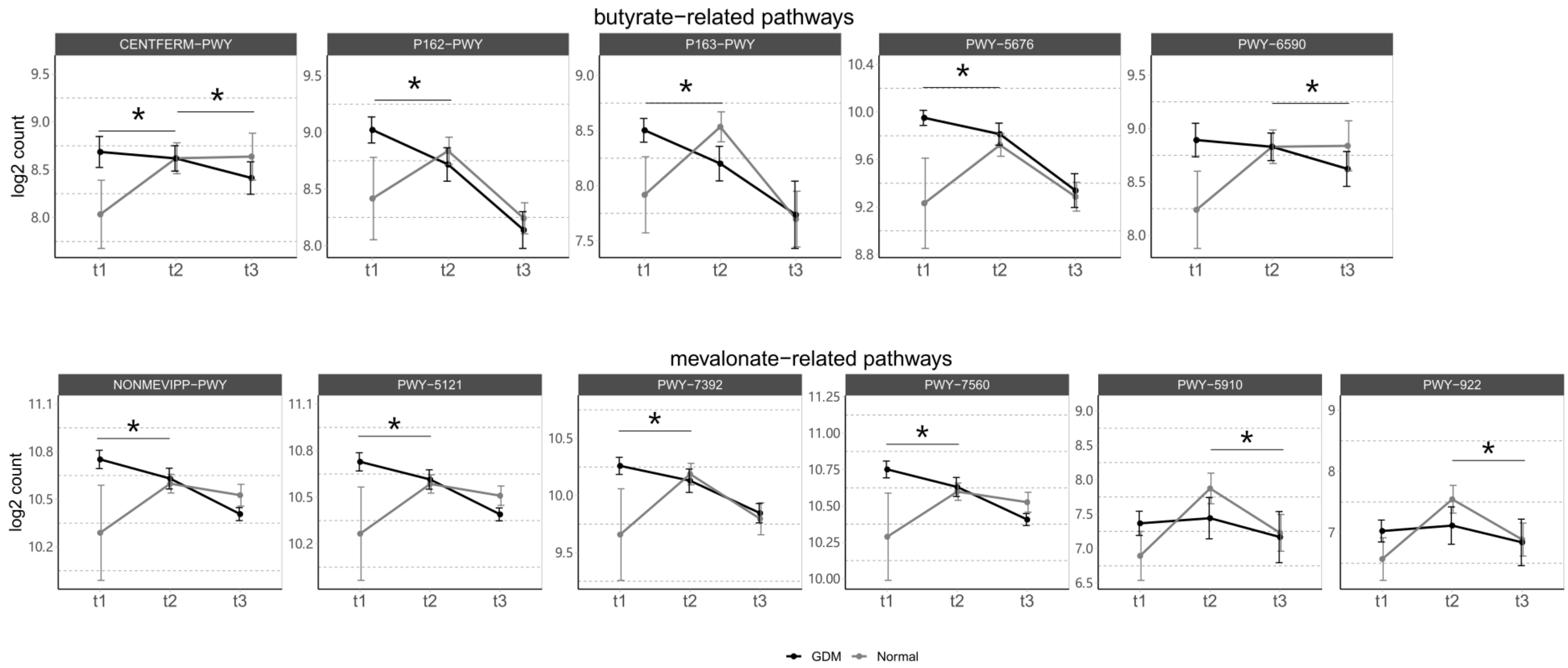

butyrate−related pathways
CENTFERM−PWY
P162−PWY
P163−PWY
PWY−5676
PWY−6590
log2 count
t1
t2
t3
mevalonate−related pathways
NONMEVIPP−PWY
PWY−5121
PWY−7392
PWY−7560
PWY−5910
PWY−922
GDM
Normal

Figure S9

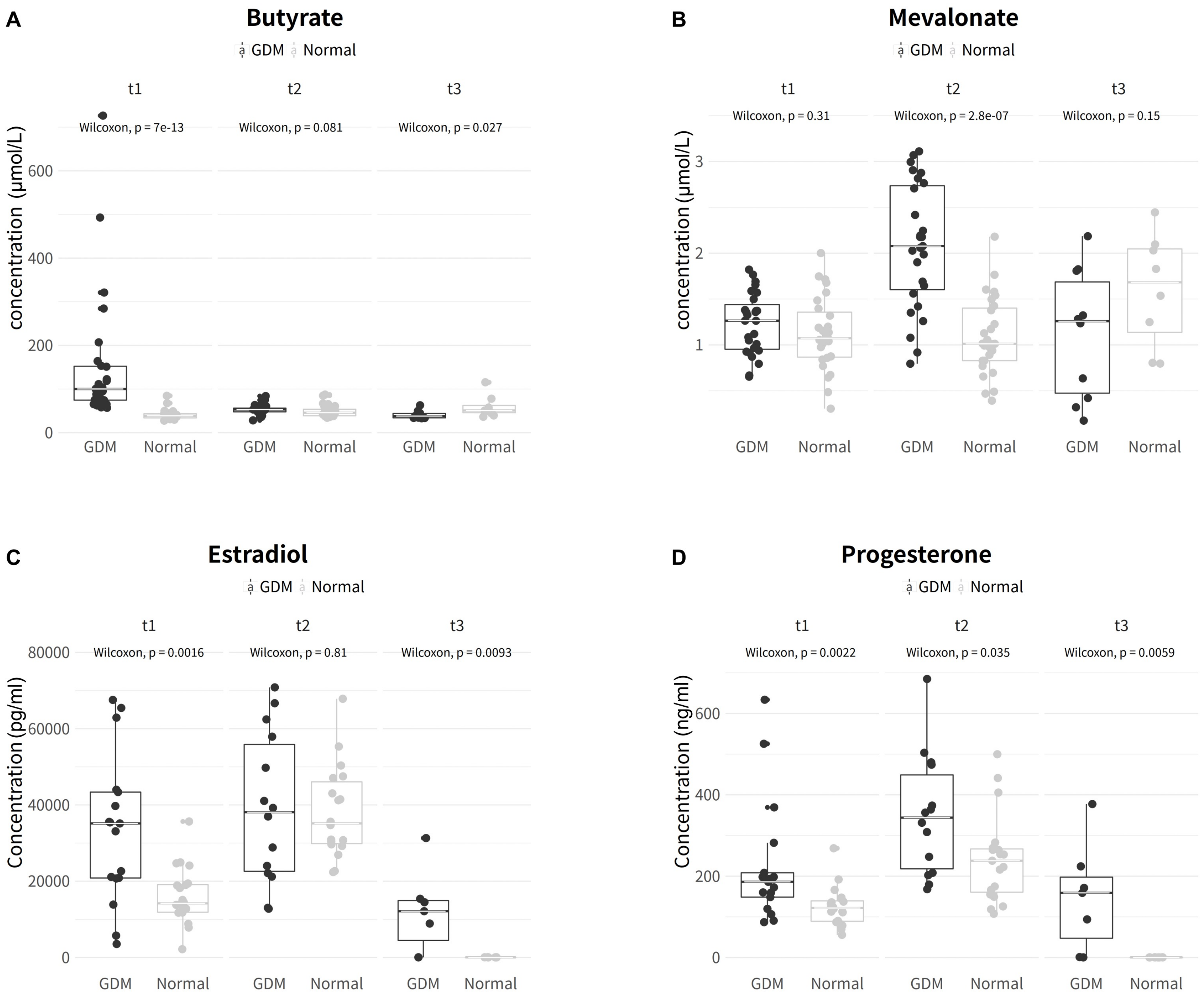
A
Butyrate
GDM Normal
t1
t2
t3
Wilcoxon, p = 7e-13
Wilcoxon, p = 0.081
Wilcoxon, p = 0.027
concentration (μmol/L)
B
Mevalonate
Wilcoxon, p = 0.31
Wilcoxon, p = 2.8e-07
Wilcoxon, p = 0.15
C
Estradiol
Wilcoxon, p = 0.0016
Wilcoxon, p = 0.81
Wilcoxon, p = 0.0093
Concentration (pg/ml)
D
Progesterone
Wilcoxon, p = 0.0022
Wilcoxon, p = 0.035
Wilcoxon, p = 0.0059
Concentration (ng/ml)

# Figure S10

A

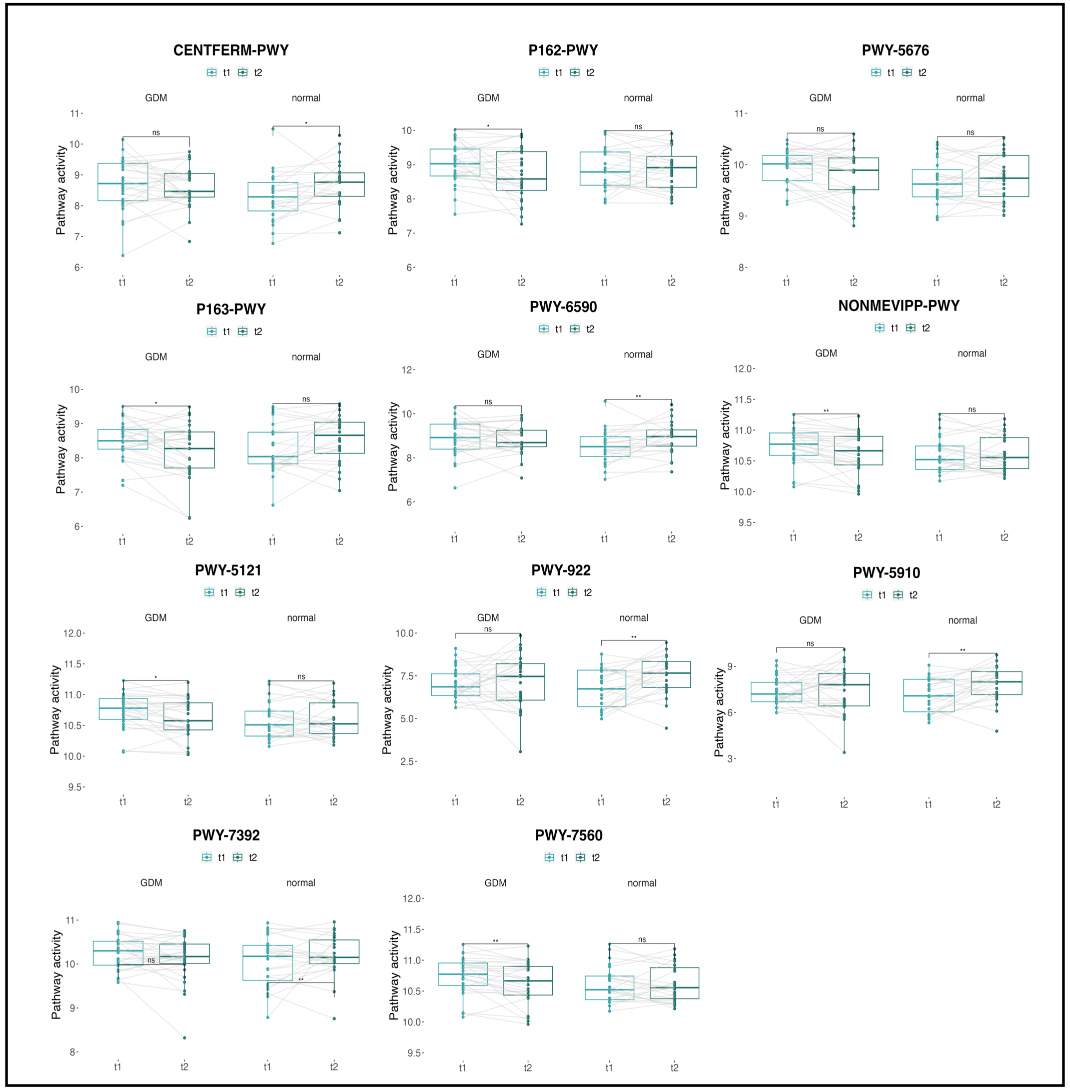


B

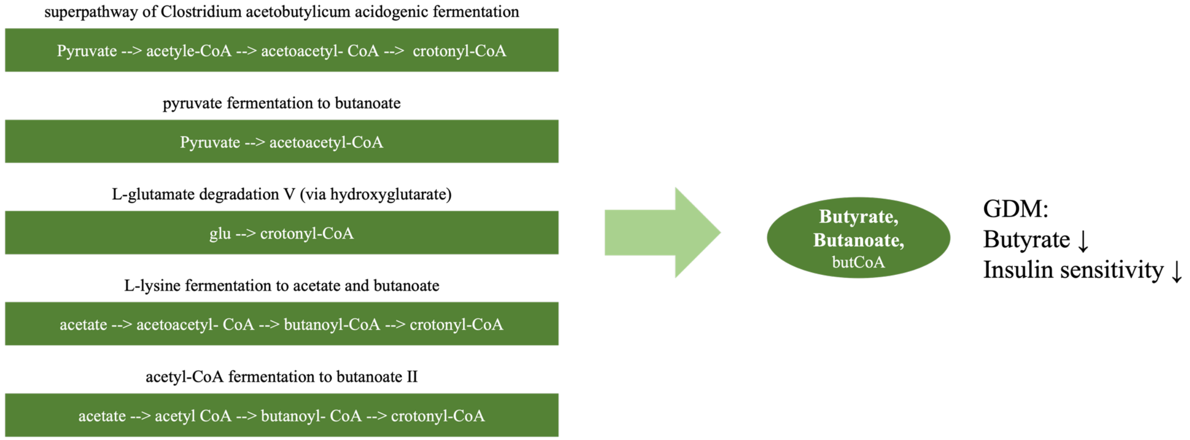


C

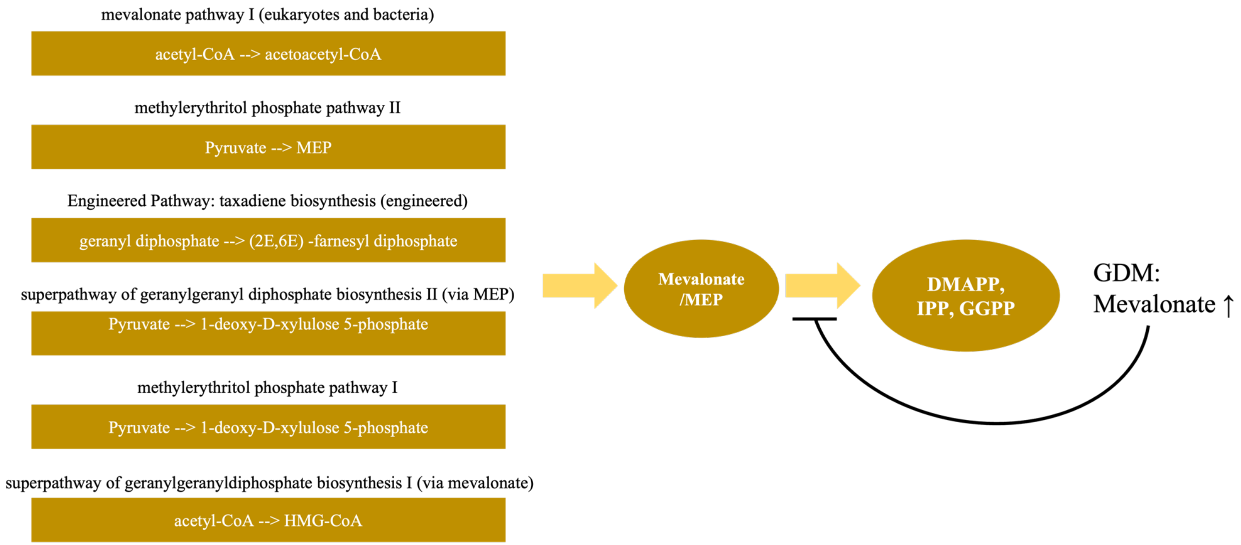

Supplement: Supplementary file 1 [file Data_Sheet_1.zip › Supplementary Figures.PDF]
